# Hierarchical Discriminative Feature Refinement for Robust and Lightweight Clothing Detection

**DOI:** 10.3390/s26216876

**Published:** 2026-10-30

**Authors:** Huawen Li, Kun Yang, Yingchao Wang, Le Chang, Feng Xue, Hui Li

**Affiliations:** 1 School of Fashion Design and Engineering, Zhejiang Sci-Tech University, Hangzhou 311199, China; 2 Graduate School of Information, Production and Systems, Waseda University, Kitakyushu 808-0135, Japan; 3 School of Intelligent Manufacturing and Electrical Engineering, Xinjiang College of Science & Technology, Korla 841000, China; 4 School of Fashion Art and Engineering, Beijing Institute of Fashion Technology, Beijing 100029, China; 5 Graduate School of Frontier Sciences, The University of Tokyo, Kashiwa 277-8561, Japan

**Keywords:** clothing detection, lightweight object detection, YOLO26n, hierarchical discriminative feature refinement, gated attention, multi-scale feature refinement, robust visual perception

## Abstract

Accurate and efficient clothing detection remains challenging because visually similar garment categories can share appearance cues, while background interference, scale variation, and spatial overlap weaken category-discriminative features. To address these issues, this study proposes a lightweight clothing detector based on YOLO26n with hierarchical discriminative feature refinement. The framework progressively enhances garment representations at the channel, semantic, and multi-scale spatial levels. Specifically, the C3k2-based Channel Feature Enhancement Module (C3k2_CFEM) strengthens cross-channel interaction and adaptively balances local details with contextual information. The C2-based Gated Semantic Attention module (C2GSA) selectively retains task-relevant high-level responses while suppressing redundant contextual interference. The Multi-Scale Dual-Stream Attention Module (MSDSAM) combines complementary foreground–background attention with efficient multi-scale feature interaction. A multi-source clothing dataset containing 11,970 images and 21,504 annotated garment instances across seven categories was used for evaluation. The proposed model achieves 91.6% Precision, 86.2% Recall, 92.6% mAP_50_, and 74.7% mAP_50:95_, improving the YOLO26n baseline by 6.6 and 5.8 percentage points in mAP_50_ and mAP_50:95_, respectively. These results are obtained with 2.39 M parameters, 5.4 billion floating-point operations (GFLOPs), and an inference speed of 166 frames per second (FPS) on an NVIDIA RTX 4090 at 640×640 input resolution and batch size 1. Additional evaluations report the model’s behavior across clothing categories, target scales, overlap levels, image perturbations, hardware platforms, and external clothing datasets. Under the reported protocols, the framework improves detection accuracy with limited increases in parameters and computational cost.

## 1. Introduction

### 1.1. Background and Motivation

Clothing detection provides garment locations and categories for visual search, recommendation, automatic annotation, and retail analysis. DeepFashion, ModaNet, DeepFashion2, and Fashionpedia established large-scale benchmarks covering consumer, shop, and street-fashion images together with detection, segmentation, retrieval, garment-part, and attribute annotations [1,2,3,4]. Reviews of visual fashion intelligence likewise identify garment localization as a foundation for downstream fashion analysis [5,6].

Transfer-learning-based surveillance models have likewise combined spatial and temporal evidence for complex visual recognition [7]. Compact fusion architectures have also been used for joint object recognition and multi-object tracking in video surveillance [8].

Garments vary substantially in style, material, viewpoint, scale, and surrounding context, while visually similar categories can share colors, textures, and silhouettes. A practical detector must therefore preserve category-discriminative garment cues without imposing an excessive computational cost.

Region-focused residual networks with squeeze-and-excitation recalibration provide a related example of preserving discriminative local evidence in domain-specific imagery [9]. Preprocessing, feature extraction, and ensemble decision strategies have likewise been combined in domain-specific image classification [10].

### 1.2. Challenges in Clothing Detection

Three representation problems motivate this study. First, visually similar clothing categories may differ mainly in localized structures such as necklines, sleeves, cuffs, and hemlines [11,12,13,14,15]; repeated convolution and downsampling can weaken these cues, especially for small targets. Second, body regions, accessories, adjacent garments, shelves, shadows, and other context can produce high-level activations that are correlated with clothing but uninformative for category discrimination. Third, scale variation increases foreground–background ambiguity because background responses occupy a larger proportion of the representation as the visible garment region becomes smaller. Feature pyramids, scale-aware receptive fields, bidirectional fusion, and attention-based refinement are established responses to scale variation and partial visibility [16,17,18,19,20].

Explicit line features have also been used to preserve geometric structure under weak texture and strong illumination [21].

These effects arise at different stages of feature extraction and fusion. The central problem is therefore to preserve subtle category cues, filter irrelevant high-level semantics, and refine scale-sensitive spatial representations within a single lightweight hierarchy.

### 1.3. Existing Methods and Research Gap

Modern detectors improve representation through feature extraction, multi-scale aggregation, contextual modeling, and efficient prediction architectures [22]. In clothing analysis, prior work has combined YOLO-based detection with transfer learning [23], introduced Transformer and receptive-field enhancements for occlusive and multi-scale clothing scenes [24], and adapted pretrained vision models with parameter-efficient modules [25]. These methods demonstrate the value of clothing-oriented feature and fusion design.

Lightweight recognition frameworks in other sensing domains have combined dynamic upsampling, gated feature interaction, and multiview fusion to retain small-target geometry under constrained computation [26]. Interaction-based fusion and uncertainty-aware auxiliary supervision have also been explored in 3D detection to preserve complementary evidence during feature aggregation [27,28]. Global–local directional aggregation provides a further example of compact YOLO-based detection for small, low-contrast targets in cluttered environments [29]. Beyond detection, attention-guided residual architectures have also been used to strengthen feature learning in image steganography [30].

Related structure-sensitive detection work has combined shape-aware processing with spatial–channel feature fusion to preserve weak geometric cues in complex backgrounds [31]. This cross-domain study provides an example of feature interaction organized around target structure.

Most existing approaches emphasize one part of the detection pipeline, whereas visual similarity, semantic interference, and scale-dependent foreground–background ambiguity affect different representation stages. YOLO26n provides a lightweight baseline for studying this interaction. YOLO26 uses native non-maximum suppression (NMS)-free inference, a lightweight detection head without Distribution Focal Loss, and a small-target-aware label assignment strategy [32]. Clothing detection requires coordinated channel interaction, semantic filtering, and foreground–background spatial refinement across this hierarchy.

This motivates a hierarchical design that strengthens subtle garment cues, selectively regulates high-level responses, and refines high-resolution features before prediction.

### 1.4. Proposed Framework and Contributions

**Figure 1:**
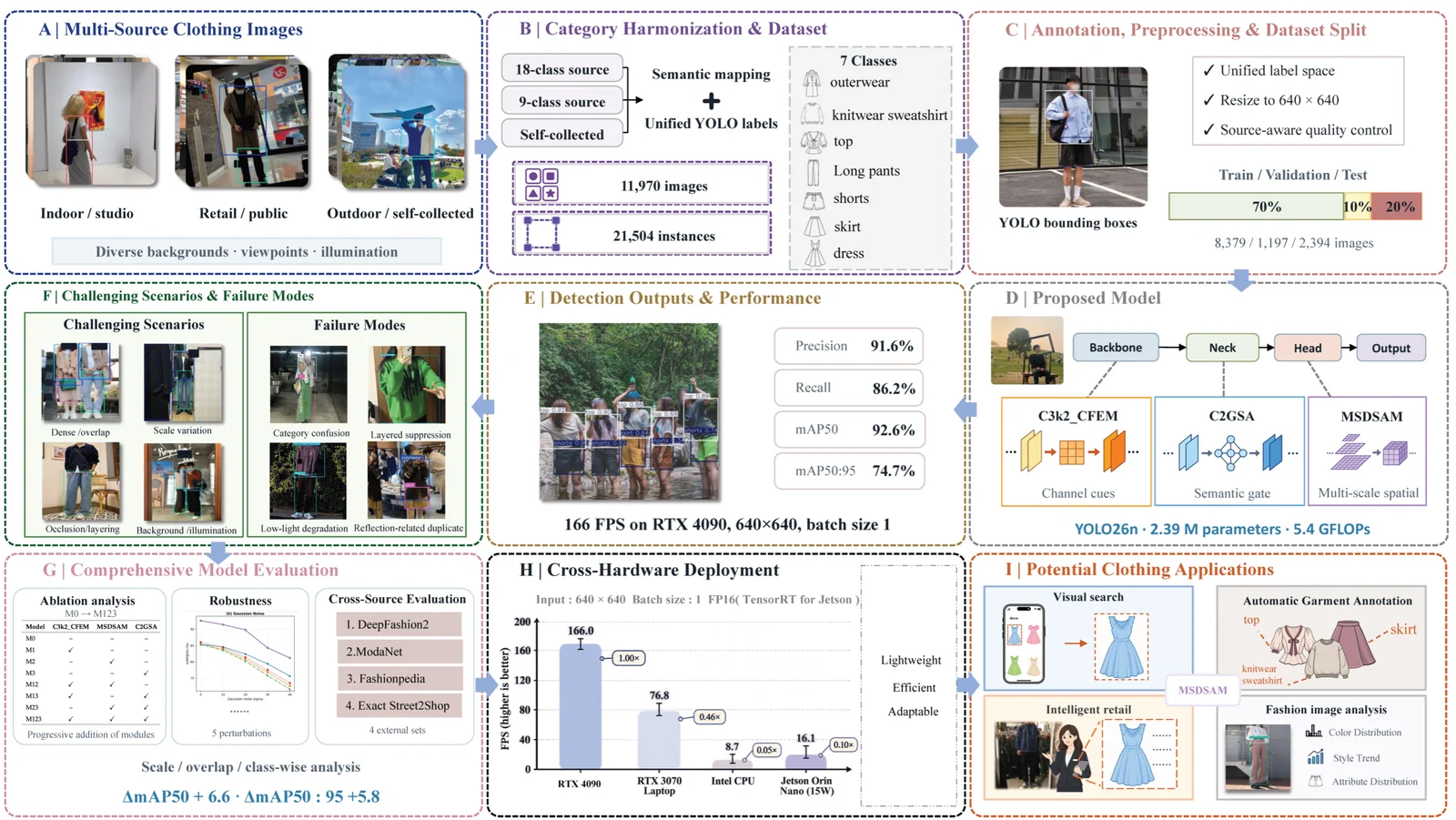
Overall framework of the proposed clothing detection system. The diagram separates category harmonization, hierarchical channel–semantic–spatial refinement using C3k2_CFEM, C2GSA, and MSDSAM, the reported evaluation protocols, and potential clothing-analysis application contexts.

To address the above limitations, this study develops a lightweight clothing detection framework based on YOLO26n through hierarchical discriminative feature refinement. C3k2_CFEM introduces progressive channel-group interaction and input-adaptive local–context fusion; C2GSA extends the C2 Position-Sensitive Attention (C2PSA) pathway with input-conditioned gating and passes the refined high-level response to MSDSAM; and MSDSAM uses this guidance to refine the high-resolution P3 feature before prediction. Together, the modules form a progressive channel–semantic–spatial pathway, summarized in Figure 1.

The main contributions of this study are summarized as follows:

- A hierarchical feature-refinement architecture is developed by redesigning C3k2 and C2PSA processing and coupling their outputs with a semantic-guided dual-stream P3 refinement stage. The three modules form an explicit channel–semantic–spatial information path.
- Under the reported five-run evaluation, the model improves mAP_50_ and mAP_50:95_ over YOLO26n by 6.6 and 5.8 percentage points, respectively, with 2.39 M parameters and 5.4 GFLOPs. Its measured throughput is 166 FPS on an NVIDIA RTX 4090 at 640×640 input resolution and batch size 1.

## 2. Materials and Methods

### 2.1. Multi-Source Dataset Construction and Category Harmonization

The final dataset combines 5776 images from the Clothes Category Dataset [33], 2483 images from the *Fashion Dataset* [34], and a 3711-image subset selected from the Street Fashion Style (SFS) dataset [35,36]. These sources account for 48.3%, 20.7%, and 31.0% of the 11,970 images, respectively. The two detection datasets hosted on Roboflow Universe (Roboflow, Inc., Des Moines, IA, USA) are distributed under the CC BY 4.0 license. SFS contains street-style outfit photographs collected from 293,105 Chictopia user posts and is provided for non-commercial research and educational use under its source terms.

The SFS subset consists of user-posted street images collected under heterogeneous consumer capture conditions. Its source metadata provide no standardized camera configuration. An image was retained when at least one directly visible garment could be assigned to the unified taxonomy and enclosed by a reliable bounding box. Corrupted files, severe blur or exposure, and cases with insufficient visible garment area for a reliable category and box were excluded. The selected images were manually annotated in YOLO bounding-box format. A second annotation pass checked category consistency, box coverage, and missed visible garments before the dataset was split.

Before multi-source integration, exact duplicate files were removed. Near-identical copies, resized or cropped versions, mirrored variants, and other transformations of the same source image were grouped within the same subset. Multiple photographs of the same garment or person were retained as distinct samples when they showed a different pose, viewpoint, or background; visually related image sequences were kept within one subset. These controls were applied before the fixed training, validation, and test split.

The two Roboflow detection datasets provide 27 source-label entries across their separate taxonomies (18 from the Clothes Category Dataset and 9 from the Fashion Dataset). These entries correspond to 23 distinct normalized label names because four labels overlap across the two sources: jacket, shirt, sweater, and the long-sleeve category, represented as long-sleeve garment in one source and long-sleeved shirt in the other. All 27 source-label entries were mapped to the seven-category taxonomy. Jacket, blazer, coat, padding, zip-up, and suit-jacket labels were mapped to outerwear; cardigan, hoodie, MTM, and sweater were mapped to knitwear_sweatshirt; and shirt, long-sleeved shirt, short-sleeved shirt, polo shirt, T-shirt, and tank top were mapped to top. Cotton pants, denim pants, slacks, and training pants were mapped to long_pants; short pants were mapped to shorts; and skirt and dress were retained. All 27 source-label entries entered the mapping, and no source class was removed. Detection ground truth for the SFS subset was created through manual YOLO annotation under the same seven-category taxonomy. Images with ambiguous garment structure after review were excluded.

The annotation target is a directly visible physical garment instance. All visible garments were annotated regardless of foreground or background location. Mirror reflections, posters, screens, and other depictions were treated as visual context and did not receive separate instance annotations. The same target definition was applied to the harmonized Roboflow annotations and the author-generated SFS annotations.

After category harmonization, the dataset contained 11,970 images and 21,504 annotated garment instances. The training, validation, and independent test subsets contained 8379, 1197, and 2394 images, with 15,032, 2133, and 4339 instances, respectively. The class counts for *outerwear*, *knitwear_sweatshirt*, *top*, *long_pants*, *shorts*, *skirt*, and *dress* were 4476, 3494, 3462, 7206, 783, 1049, and 1034.

**Figure 2:**
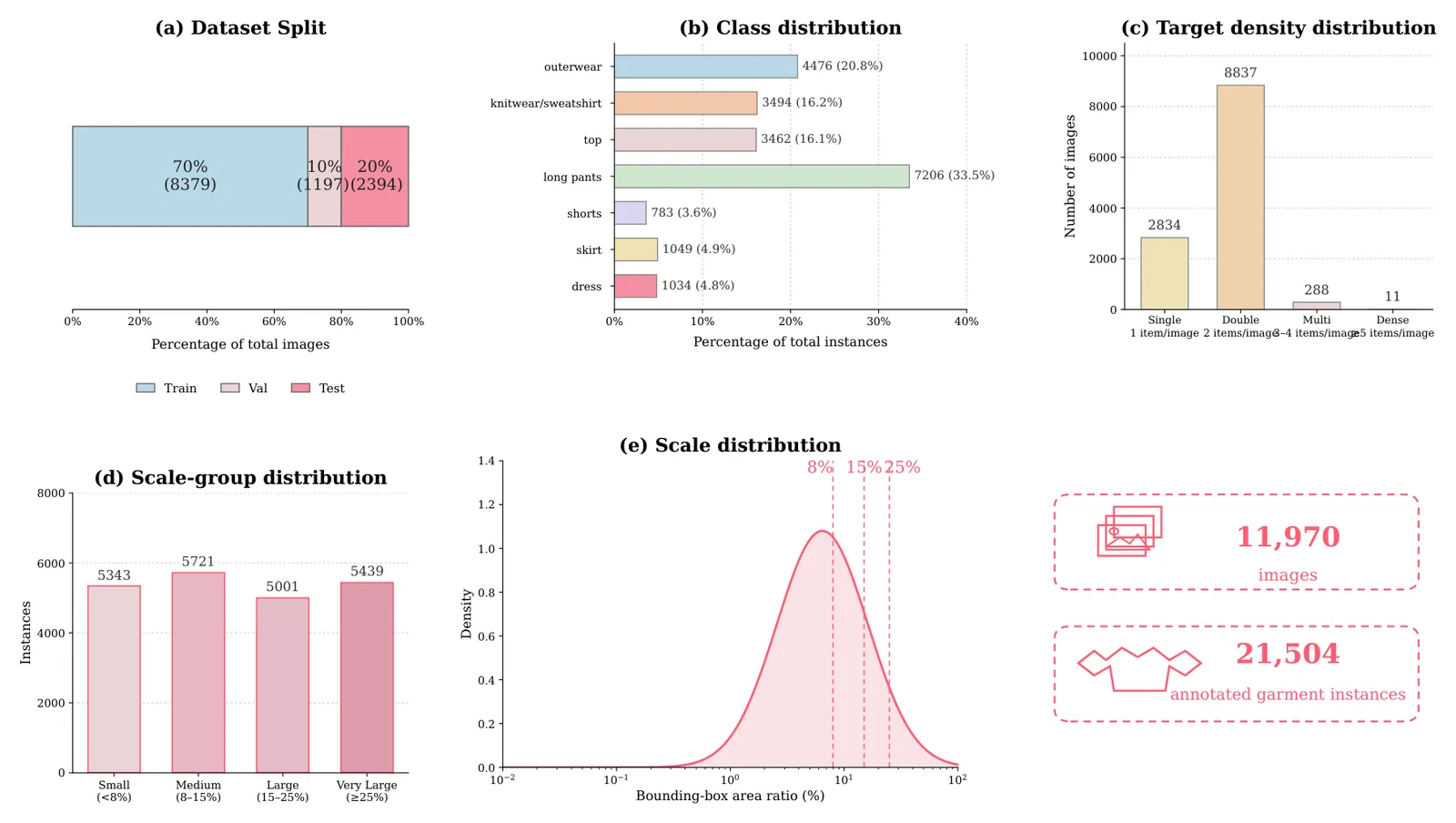
Statistical overview of the constructed clothing detection dataset: (**a**) dataset split, (**b**) class distribution, (**c**) image-level target density distribution, (**d**) instance distribution across four scale groups, and (**e**) continuous distribution of the relative bounding-box area.

Figure 2 summarizes the dataset split, class composition, image-level target density, and bounding-box scale distribution. These statistics characterize variation in category, target density, object scale, background, appearance, and imaging condition within a consistent annotation space.

### 2.2. Overall Architecture of the Proposed Model

**Figure 3:**
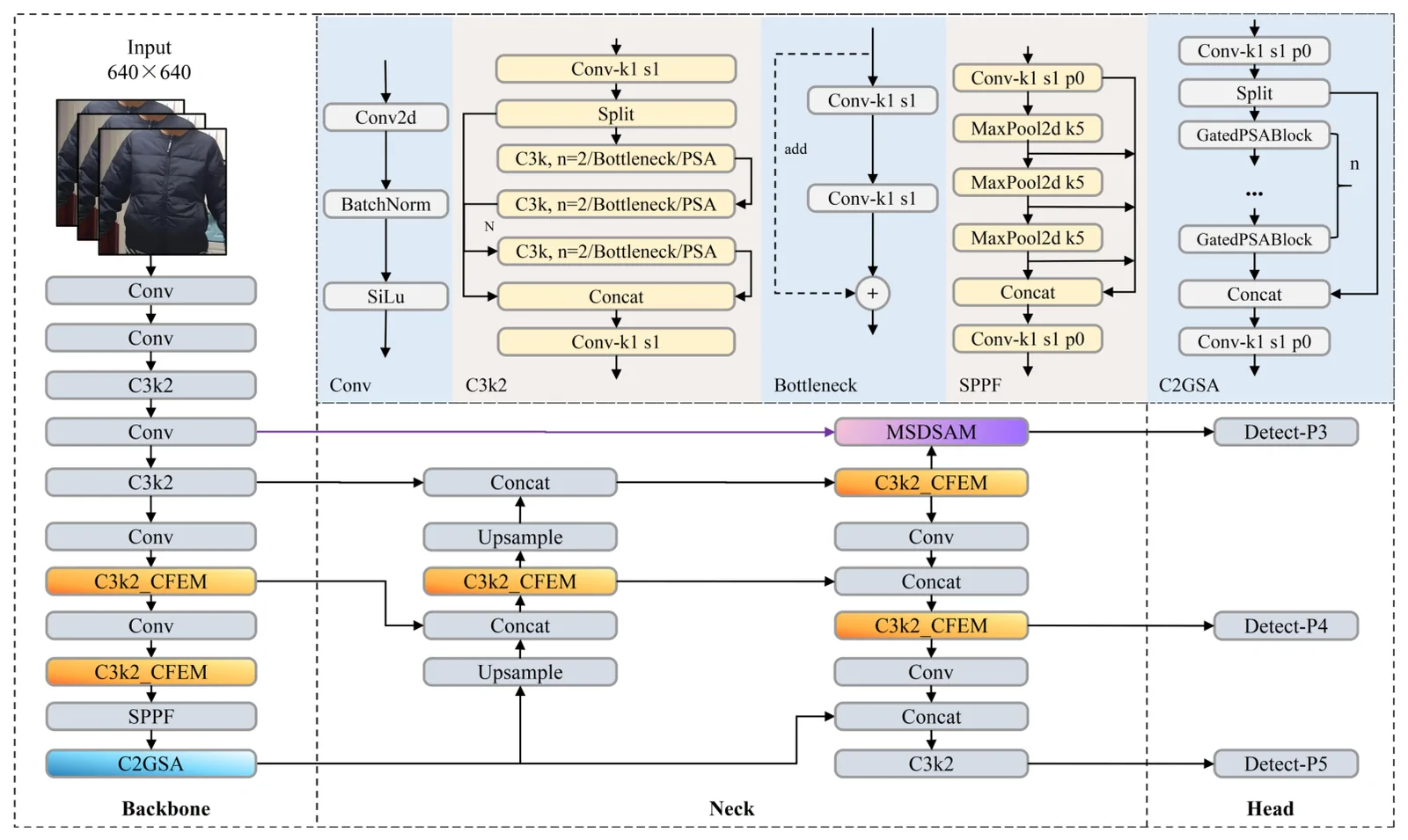
Overall architecture of the proposed clothing detection model. Five C3k2_CFEM blocks are used: two in the deeper backbone stages and three in the neck. C2GSA refines the terminal high-level semantic feature, and MSDSAM performs semantic-guided foreground–background refinement immediately before the P3 detection branch.

The proposed clothing detector is developed on the lightweight multi-scale detection framework of YOLO26n and preserves its backbone–neck–head organization. As shown in Figure 3, the network contains a hierarchical backbone for feature extraction, a neck for multi-scale aggregation, and detection heads operating on the P3, P4, and P5 feature levels. The design modifies three critical feature-processing stages according to the distinct representation requirements of clothing detection.

First, C3k2_CFEM is embedded at the five C3k2 stages shown in Figure 3: two deeper stages of the backbone and three stages of the neck. These placements cover the feature levels at which local garment cues are first consolidated and then repeatedly fused across scales. The two backbone blocks strengthen the deeper feature basis before Spatial Pyramid Pooling–Fast (SPPF) and semantic attention. In the neck, one block follows the first top-down fusion, one refines the high-resolution P3 fusion before MSDSAM, and one refines the subsequent bottom-up P4 fusion; the final P5 branch retains the standard C3k2 block. This selective arrangement extends channel-group interaction across both feature extraction and bidirectional aggregation while limiting additional complexity.

Second, the terminal C2PSA stage of the backbone is replaced by C2GSA after SPPF. C2GSA introduces an input-conditioned element-wise gate into the self-attention pathway, allowing the network to regulate which high-level responses propagate after global dependency modeling. Its placement at the terminal backbone stage provides a semantically consolidated feature for neck fusion and for the subsequent spatial refinement stage.

Third, MSDSAM is inserted immediately before the P3 detection branch, as shown in Figure 3. It combines the gated high-level semantic signal from C2GSA with the high-resolution P3 feature. The semantic feature generates complementary foreground- and background-priority responses, which reorganize and refine the P3 representation through parallel multi-scale streams before it is forwarded to the P3 detection head. The P4 and P5 branches retain their corresponding detection paths.

The resulting order follows the information hierarchy shown in Figure 3. C3k2_CFEM first strengthens the channel basis during extraction and fusion, C2GSA then filters the terminal high-level semantic response, and MSDSAM finally uses that semantic response to refine the high-resolution spatial feature. This explicit channel–semantic–spatial dependency explains the selected positions, number, and order of the modules while retaining the lightweight organization of YOLO26n.

### 2.3. Key Modules of the Proposed Model

#### 2.3.1. Channel-Aware C3k2_CFEM Module

Visually similar clothing categories frequently share global appearance while differing in localized texture, boundary shape, or structural details. C3k2_CFEM therefore combines progressive channel-group interaction with adaptive local–context fusion within the C3k2 transformation.

**Figure 4:**
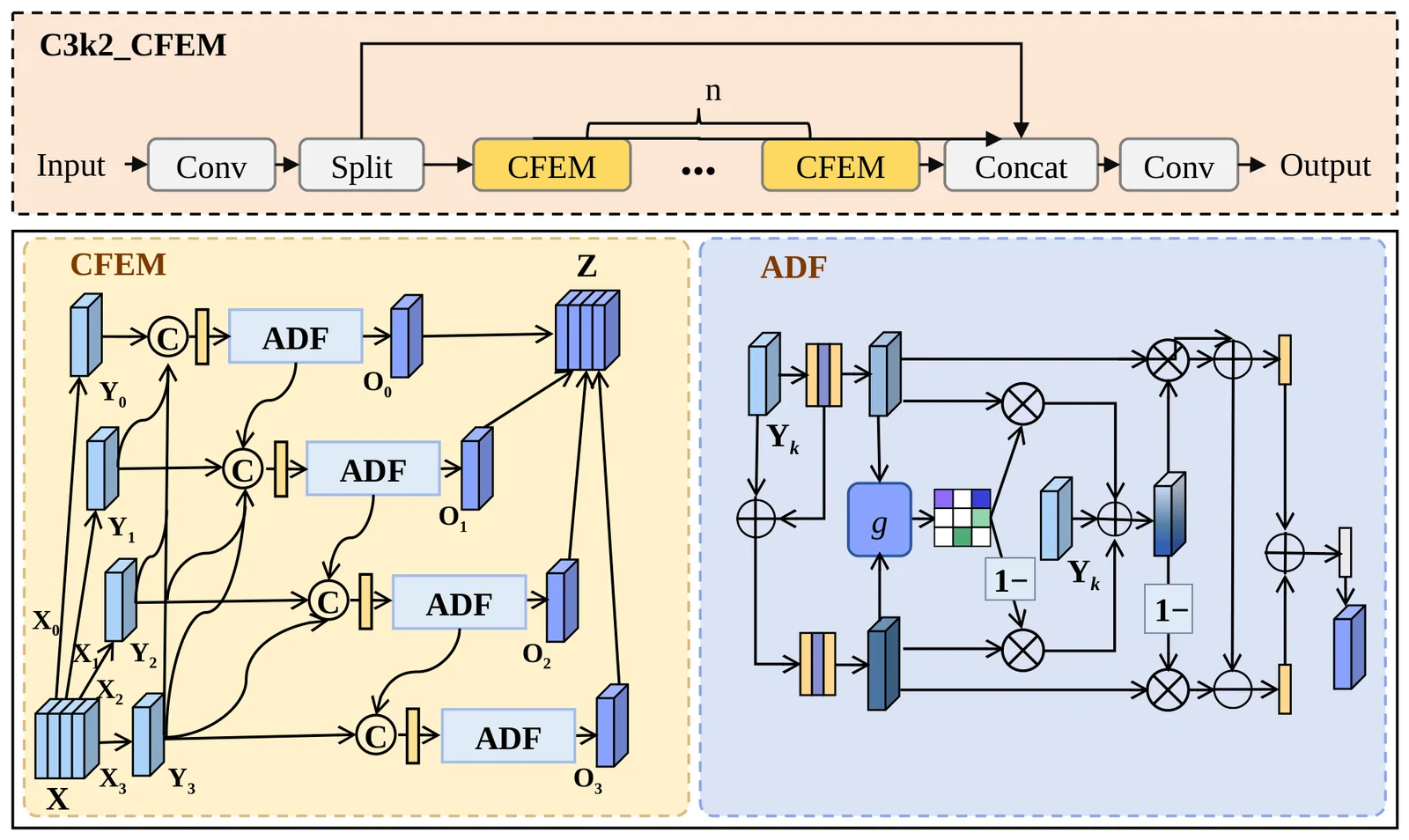
Structure of the proposed C3k2_CFEM module. The circled “C” denotes channel-wise concatenation, and the projection following each concatenation restores the stable group dimension before ADF processing. In the ADF inset, $Y_{k}$, $Y_{A}$, $Y_{B}$, and *g* correspond to $P_{k}$, $F_{1}$, $F_{2}$, and α, respectively, and the rightmost output corresponds to $O_{k}$. Progressive channel-group interaction is combined with adaptive dynamic fusion to preserve category-discriminative garment representations.

As shown in Figure 4, given an input feature $X\in R^{B\times C\times H\times W}$, it is first divided into four groups along the channel dimension:

(1) $$X=\left[X_{0},X_{1},X_{2},X_{3}\right],\;X_{k}\in R^{B\times \frac{C}{4}\times H\times W}.$$

The circled “C” symbols in Figure 4 denote channel-wise concatenation. Each split group is first transformed into a group feature,

$$Y_{k}=\eta_{k}\left(X_{k}\right),\;k\in \left\{0,1,2,3\right\}.$$

where $\eta_{k}\left(\cdot \right)$ denotes the group-specific convolutional transformation shown in the diagram. Progressive interaction is then formed by concatenating the current and subsequent group features together with the enhanced output of the preceding stage:

(2) $$P_{k}=\left\{\begin{array}{cc} \pi_{0}\;\left(\mathrm{Cat}(Y_{0},Y_{1},Y_{2},Y_{3})\right), & k=0,\\ \pi_{k}\;\left(\mathrm{Cat}(Y_{k},\ldots ,Y_{3},O_{k-1})\right), & k\in \{1,2,3\}, \end{array}\right.$$

where $O_{k-1}$ is the enhanced output of the preceding stage and $\pi_{k}\left(\cdot \right)$ denotes the projection immediately following each concatenation in Figure 4. This projection maps the variable-width concatenated tensor back to $R^{B\times \frac{C}{4}\times H\times W}$ before ADF processing. Consequently, cross-group information is propagated through concatenation while every ADF block operates on a stable tensor dimension.

In the ADF inset of Figure 4, $Y_{k}$, $Y_{A}$, $Y_{B}$, and *g* correspond to $P_{k}$, $F_{1}$, $F_{2}$, and α, respectively, in Equations (2)–(5); the rightmost output feature corresponds to $O_{k}$.

To further control the balance between localized detail and contextual representation, an Adaptive Dynamic Fusion (ADF) unit is introduced within each group. Two sequential nonlinear transformations are first constructed as

(3) $$F_{1}=\phi_{1}\left(P_{k}\right),\;F_{2}=\phi_{2}\left(P_{k}+F_{1}\right),$$

where $\phi_{1}$ and $\phi_{2}$ consist of convolution and nonlinear activation. The two responses are subsequently used to predict a channel-dependent fusion coefficient:

(4) $$\alpha =\psi \left(\mathrm{Cat}(F_{1},F_{2})\right),\;\alpha \in \left(0,1\right),$$

where ψ(·) combines global average pooling and max pooling followed by shared convolution and sigmoid activation. The output of each group is then obtained by

(5) $$O_{k}=P_{k}+\alpha ⊙F_{1}+\left(1-\alpha \right)⊙F_{2}.$$

This formulation allows the relative contribution of the two feature transformations to vary with the input rather than being fixed throughout the network. Finally, the four enhanced groups are concatenated and projected by a 1×1 convolution:

(6) $$Z={\mathrm{Conv}}_{1\times 1}\left(\mathrm{Cat}(O_{0},O_{1},O_{2},O_{3})\right).$$

The resulting C3k2_CFEM differs from a conventional grouped transformation in two aspects: cross-group information is propagated recursively, and the local–context contribution within each group is dynamically determined from the input feature.

#### 2.3.2. Gated Semantic Attention C2GSA Module

High-level self-attention captures long-range dependencies but may preserve target-relevant semantics together with visually correlated interference. C2GSA therefore adds input-conditioned gating to the C2PSA attention pathway to regulate the information propagated after global dependency modeling.

**Figure 5:**
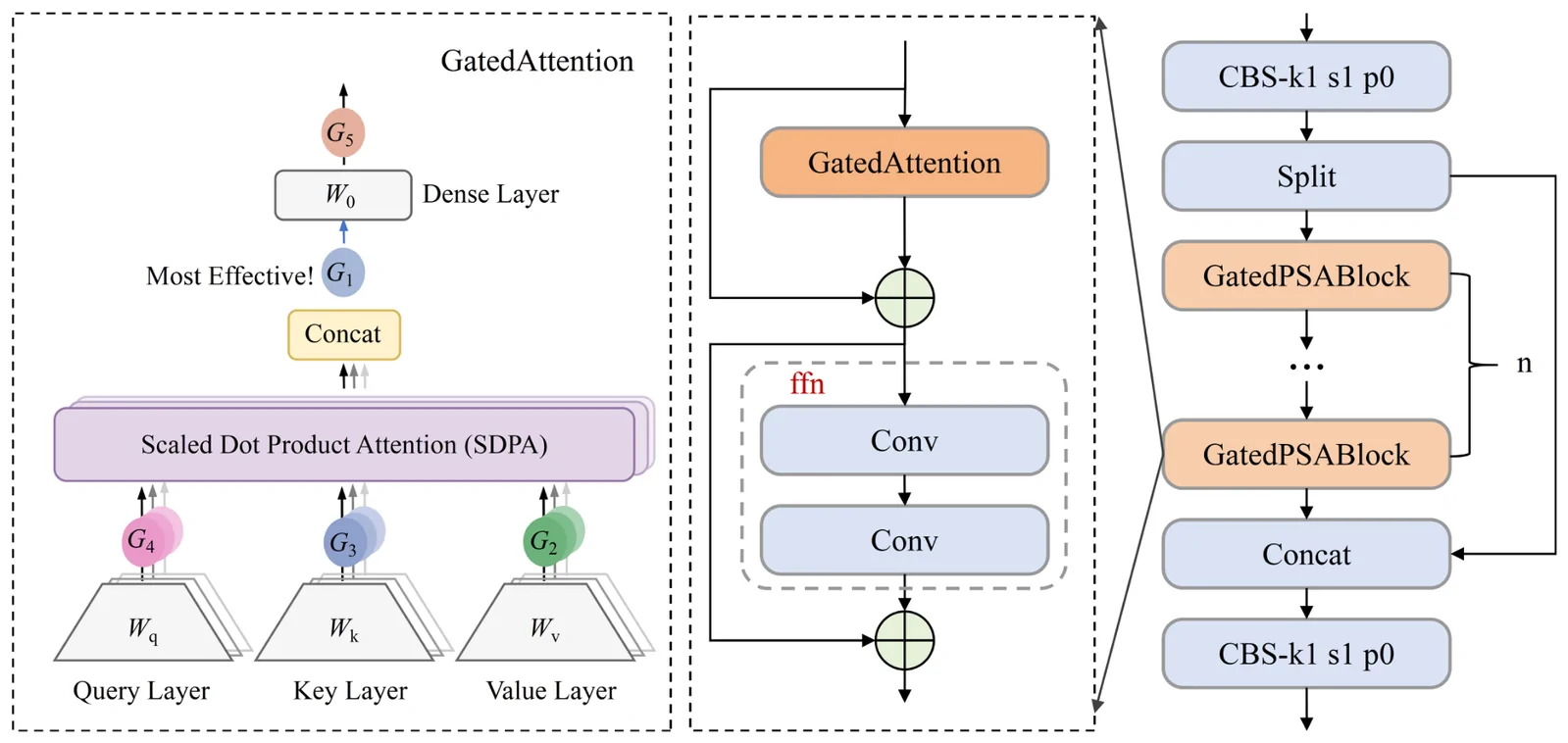
Gating principle and implementation of the proposed C2GSA module. The left schematic illustrates the general post-attention gating concept following Qiu et al. [37], whereas the right schematic shows the actual C2GSA implementation for visual feature maps, in which query, key, value, and gating representations are generated using convolutional projections. In the right schematic, CBS-k1 s1 p0 denotes a convolution–batch-normalization–SiLU projection with kernel size 1, stride 1, and padding 0; ffn denotes the convolutional feed-forward network; and GatedPSABlock denotes the repeated gated-attention–FFN unit.

Figure 5 contrasts the general post-attention gating formulation reported by Qiu et al. [37] with the C2GSA implementation used in this study. The left schematic summarizes the generic query–key–value and dense-layer formulation, whereas the right schematic shows convolutional attention and gating projections on two-dimensional visual feature maps.

In Figure 5, CBS-k1 s1 p0 denotes a convolution–batch-normalization–SiLU block (CBS), where SiLU is the sigmoid linear unit and k1, s1, and p0 indicate a 1×1 kernel, stride 1, and padding 0, respectively. The label ffn denotes the convolutional feed-forward network (FFN), and GatedPSABlock denotes the repeated C2PSA processing unit that combines the gated-attention sublayer with this FFN.

Given an input feature $X\in R^{B\times C\times H\times W}$, LayerNorm is first applied to obtain the normalized visual representation $X_{n}$. Query, key, and value features are then generated using three independent 1×1 convolutions:

(7) $$Q=f_{q}\left(X_{n}\right),\;K=f_{k}\left(X_{n}\right),\;V=f_{v}\left(X_{n}\right),$$

where $f_{q}\left(\cdot \right)$, $f_{k}\left(\cdot \right)$, and $f_{v}\left(\cdot \right)$ denote the corresponding 1×1 convolutional projections. After reshaping the projected features into multiple attention heads, scaled dot-product attention is computed as

(8) $$A=\mathrm{Softmax}\left(\frac{QK^{T}}{\sqrt{d_{h}}}\right),\;Y=AV,$$

where $d_{h}$ denotes the feature dimension of each attention head.

The distinguishing component of C2GSA is a parallel semantic gating branch constructed directly from the normalized visual feature. The branch predicts an element-wise control map as

(9) $$G=\sigma \left(f_{g}\left(X_{n}\right)\right),$$

where $f_{g}\left(\cdot \right)$ denotes a 1×1 convolution and σ(·) is the sigmoid function. The globally aggregated attention response is then selectively recalibrated by

(10) $$\tilde{Y}=Y⊙G,\;Z=f_{o}\left(\tilde{Y}\right),$$

where ⊙ denotes element-wise multiplication and $f_{o}\left(\cdot \right)$ is the output projection implemented using a 1×1 convolution.

C2GSA adapts post-attention gating to two-dimensional visual feature maps through convolutional attention and gating projections compatible with the C2PSA hierarchy. Its gated output also serves as the high-level semantic guidance signal for MSDSAM, directly connecting global semantic modeling with the subsequent high-resolution spatial refinement stage.

#### 2.3.3. Multi-Scale Dual-Stream Attention Module

MSDSAM uses semantic guidance to decompose the high-resolution P3 representation into complementary foreground- and background-priority streams, reducing spatially competing responses before prediction.

**Figure 6:**
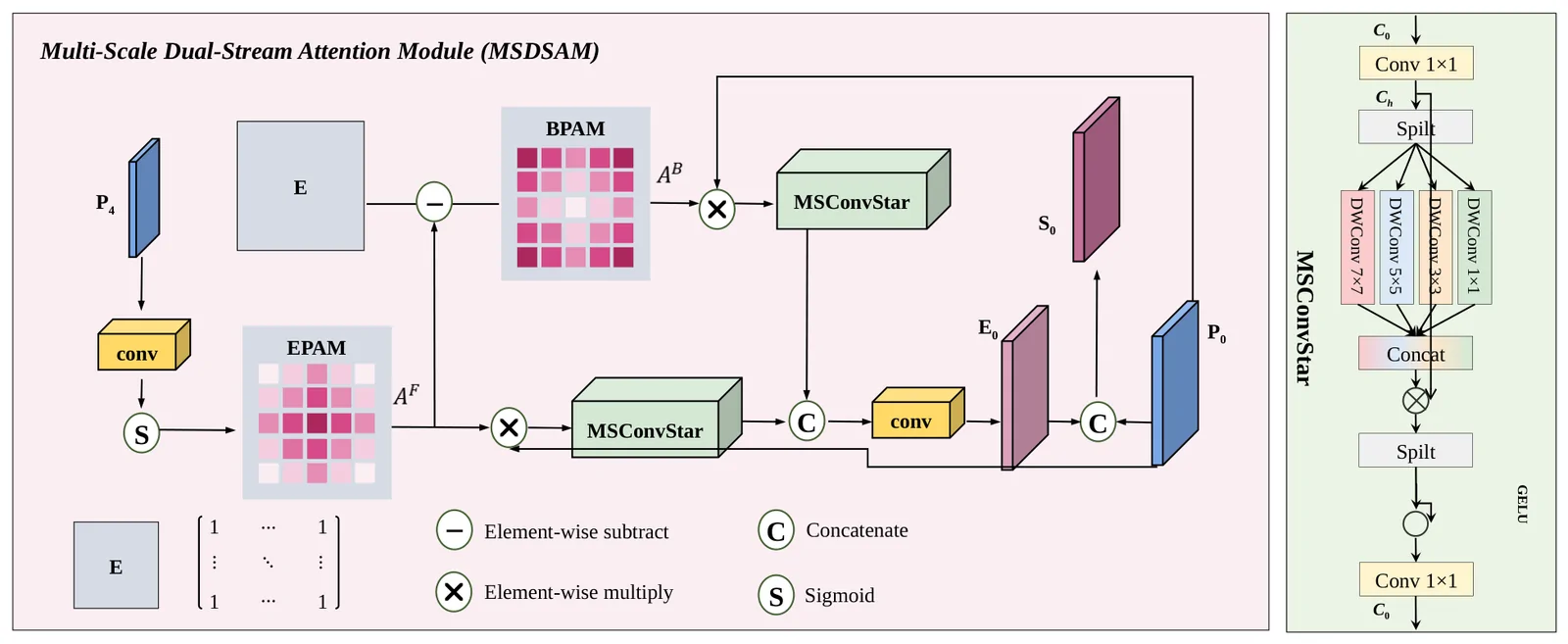
Structure and information flow of the proposed MSDSAM module. The implementation labels $P_{4}$ and $P_{0}$ correspond to $X_{h}$ and $X_{l}$; EPAM and BPAM correspond to $A^{F}$ and $A^{B}$; and $E_{0}$ and $S_{0}$ correspond to $E_{0}$ and S. MSConvStar denotes the stream-wise multi-scale and multiplicative transformation defined in the text.

Figure 6 illustrates the complete information flow of MSDSAM. Here, $X_{h}$ denotes the high-level semantic feature generated by C2GSA, whereas $X_{l}$ denotes the high-resolution P3 spatial feature from the neck. The superscripts *F* and *B* represent foreground- and background-priority attention, respectively, while the subscripts *f* and *b* distinguish the corresponding foreground and background feature streams. Throughout this subsection, σ(·) denotes sigmoid activation, Up(·) denotes bilinear upsampling, Cat(·) denotes channel-wise concatenation, DWConv denotes depthwise convolution, and ⊙ denotes element-wise multiplication.

In Figure 6, the inputs $P_{4}$ and $P_{0}$ correspond to $X_{h}$ and $X_{l}$, respectively. The block *E* denotes the all-ones tensor 1, and the circled “S” denotes the sigmoid operation σ(·). EPAM and BPAM represent the foreground- and background-priority maps $A^{F}$ and $A^{B}$. The features after the two element-wise multiplication nodes are $F_{f}$ and $F_{b}$; the two MSConvStar blocks implement the multi-scale convolution and multiplicative interaction described in Equations (13) and (14), producing $O_{f}$ and $O_{b}$. The intermediate label $E_{0}$ denotes the projected fusion of these two stream outputs, and $S_{0}$ denotes the final output S after fusion with the original $P_{0}$ pathway.

MSDSAM first converts $X_{h}$ into a foreground-priority attention map $A^{F}$. Its complementary background-priority map $A^{B}$ is then obtained by

(11) $$A^{F}=\sigma \left({\mathrm{Conv}}_{1\times 1}\left(X_{h}\right)\right),\;A^{B}=1-A^{F}.$$

Here, $A^{F}$ represents the semantic prior assigned to foreground-related responses, whereas $A^{B}$ represents its complementary background prior. Defining the two maps complementarily constrains foreground enhancement and background modeling within a common semantic partition rather than estimating them independently.

After spatial alignment with $X_{l}$, the two attention maps are applied to the P3 feature to obtain the initial foreground and background streams, denoted by $F_{f}$ and $F_{b}$, respectively:

(12) $$F_{f}=X_{l}⊙\mathrm{Up}\left(A^{F}\right),\;F_{b}=X_{l}⊙\mathrm{Up}\left(A^{B}\right).$$

The high-level semantic representation generates two soft weighting maps that reorganize the native P3 responses into complementary streams. Bilinear interpolation aligns these maps to the P3 grid, while the original P3 tensor continues to carry local boundaries and fine geometry. Element-wise modulation, multi-scale depthwise processing, and the native P3 skip pathway all operate at P3 resolution. Boundary information is therefore preserved by the native high-resolution feature path, and the semantic maps control response allocation during refinement.

Each stream is subsequently processed by a lightweight multi-scale convolutional transformation to capture clothing structures with different spatial extents. Let $F^{\left(i\right)}$ denote the *i*-th channel group obtained after channel expansion and grouping of either $F_{f}$ or $F_{b}$. The corresponding multi-scale response ${\tilde{F}}^{\left(i\right)}$ is computed as

(13) $${\tilde{F}}^{\left(i\right)}={\mathrm{DWConv}}_{k_{i}\times k_{i}}\left(F^{\left(i\right)}\right),\;k_{i}\in \left\{1,3,5,7\right\}.$$

Here, $k_{i}$ denotes the kernel size assigned to the *i*-th channel group. The use of multiple depthwise kernels provides different effective receptive fields while limiting the additional computational cost. The resulting multi-scale responses are concatenated and combined with a residual connection to form an intermediate representation.

This intermediate feature is subsequently divided into two branches, $Z_{1}$ and $Z_{2}$. Their multiplicative nonlinear interaction produces the refined representation Z:

(14) $$Z=\mathrm{GELU}\left(Z_{1}\right)⊙Z_{2},$$

where GELU(·) denotes the Gaussian Error Linear Unit. $Z_{1}$ provides a nonlinear response, whereas $Z_{2}$ serves as a content-dependent modulation branch. Their element-wise interaction enables adaptive feature modulation without substantially increasing model complexity.

The same refinement process is applied independently to the foreground and background streams. Their outputs are denoted by $O_{f}$ and $O_{b}$, respectively. The two complementary representations are first concatenated and projected to obtain the intermediate fused feature $E_{0}$. This feature is then concatenated with the original high-resolution feature $X_{l}$ to obtain the final MSDSAM output S:

(15) $$\begin{array}{cc} E_{0}\; & =\mathrm{ReLU}\left[\mathrm{BN}\left({\mathrm{Conv}}_{3\times 3}\left(\mathrm{Cat}(O_{f},O_{b})\right)\right)\right],\\ S\; & =\mathrm{Cat}(E_{0},X_{l}). \end{array}$$

where BN(·) and ReLU(·) denote batch normalization and ReLU activation, respectively. The intermediate $E_{0}$ corresponds to the label $E_{0}$ in Figure 6. Its final concatenation with the original high-resolution feature $X_{l}$ corresponds to the $P_{0}$-to-$S_{0}$ path in the schematic. Thus, S, shown as $S_{0}$ in the figure, represents the final foreground–background-refined P3 feature.

The fused output S is forwarded to the P3 detection branch, establishing a direct dependency from the Layer-10 C2GSA semantic feature to the Layer-16 high-resolution P3 feature.

### 2.4. Experimental Setup and Evaluation Metrics

All experiments were conducted under a consistent hardware and software environment. The experimental platform is summarized in Table 1. All models were implemented in PyTorch. YOLO26n and its variants, including the proposed model, were developed using the Ultralytics framework, whereas comparison detectors from other architectural families were trained using their corresponding official or widely adopted PyTorch implementations.

For the controlled experiments involving YOLO26n and the proposed model variants, identical training settings were used to isolate the contribution of the proposed modules. The input resolution was set to 640×640 pixels, and each model was trained for 200 epochs with a batch size of 16. Stochastic Gradient Descent (SGD) was used with an initial learning rate of 0.001 and a final learning-rate factor of 0.01. Eight data-loading workers were used.

Comparisons across heterogeneous detector architectures used the same dataset split, unified category taxonomy, data partitions, and evaluation protocol. Each detector followed the optimization recipe of its official or recommended implementation, including the optimizer, learning-rate schedule, data augmentation, loss function, and prediction mechanism. Hyperparameters were selected using the training and validation data, and the test set was reserved for final evaluation.

All detector comparisons reported in the main accuracy, efficiency, and accuracy–throughput analyses use a common input resolution of 640×640. The dataset split, category taxonomy, input resolution, and evaluation protocol are fixed across models, while architecture-specific optimization recipes follow the official or recommended implementations. Throughput is measured with batch size 1 under the common conditions described in Section 3.8.

Detection performance was evaluated using Precision (*P*), Recall (*R*), mAP_50_, and mAP_50:95_. Precision and Recall are defined as

(16) $$P=\frac{TP}{TP+FP},$$

(17) $$R=\frac{TP}{TP+FN},$$

where TP, FP, and FN denote true positives, false positives, and false negatives, respectively. Precision measures the proportion of correct predictions, whereas Recall reflects the ability of the detector to identify ground-truth clothing instances.

The Average Precision (AP) for the *i*-th category is calculated from the precision–recall curve as

(18) $$AP_{i}=\int_{0}^{1}P_{i}\left(R_{i}\right)\;dR_{i},$$

and the mean Average Precision is defined as

(19) $$mAP=\frac{1}{N}\sum_{i=1}^{N}AP_{i},$$

where *N* denotes the number of clothing categories. In this study, mAP_50_ represents the mean AP at an Intersection over Union (IoU) threshold of 0.50, whereas mAP_50:95_ denotes the mean AP averaged over IoU thresholds from 0.50 to 0.95 with an interval of 0.05.

In addition to detection accuracy, model efficiency was evaluated using the number of parameters (Params), floating-point operations (FLOPs), and frames per second (FPS). Model size is retained only in the controlled YOLO26n ablation, where all variants use the same framework and serialization format; it is not used for direct comparison across heterogeneous architectures.

## 3. Experimental Results

### 3.1. Ablation and Component Analysis

To assess the individual effectiveness and complementarity of C3k2_CFEM, MSDSAM, and C2GSA, a combinatorial ablation study was conducted. M0 denotes the original YOLO26n baseline; M1, M2, and M3 incorporate the three modules individually; M12, M13, and M23 represent their pairwise combinations; and M123 denotes the complete model. All variants were trained and evaluated under the same experimental protocol. Detection metrics are reported as mean ± standard deviation over repeated runs.

Table 2 shows complementary effects across the three modules. Among the single-module variants, C3k2_CFEM (M1) gives the largest Precision and mAP_50_ gains (4.2 and 3.8 percentage points), MSDSAM (M2) gives the largest Recall gain (3.6 points), and C2GSA (M3) gives the highest single-module mAP_50:95_ (71.2%). The pairwise variants improve multiple metrics, while the complete M123 model reaches 91.6% Precision, 86.2% Recall, 92.6% mAP_50_, and 74.7% mAP_50:95_, corresponding to gains of 9.3, 6.1, 6.6, and 5.8 percentage points over YOLO26n.

Internal-mechanism factorial ablation. To distinguish the empirical contributions of the principal internal components, a $2^{3}$ factorial ablation was conducted over the Adaptive Dynamic Fusion (ADF) unit in C3k2_CFEM, the semantic gate in C2GSA, and the foreground–background dual-stream mechanism in MSDSAM. Each factor was switched on or off, while the remaining block structure and the five-run training protocol were kept unchanged. A0 serves as the internal-factor reference configuration.

As shown in Table 3, ADF alone (A1) produces the largest single-factor gains in Precision and mAP_50_ (1.68 and 1.24 percentage points), the semantic gate alone (A2) produces the largest mAP_50:95_ gain (1.54 points), and the dual-stream mechanism alone (A3) produces the largest Recall gain (1.44 points). The complete A7 configuration achieves the highest value for all four metrics and exceeds A0 by 4.10, 2.50, 3.20, and 3.40 percentage points, respectively.

**Figure 7:**
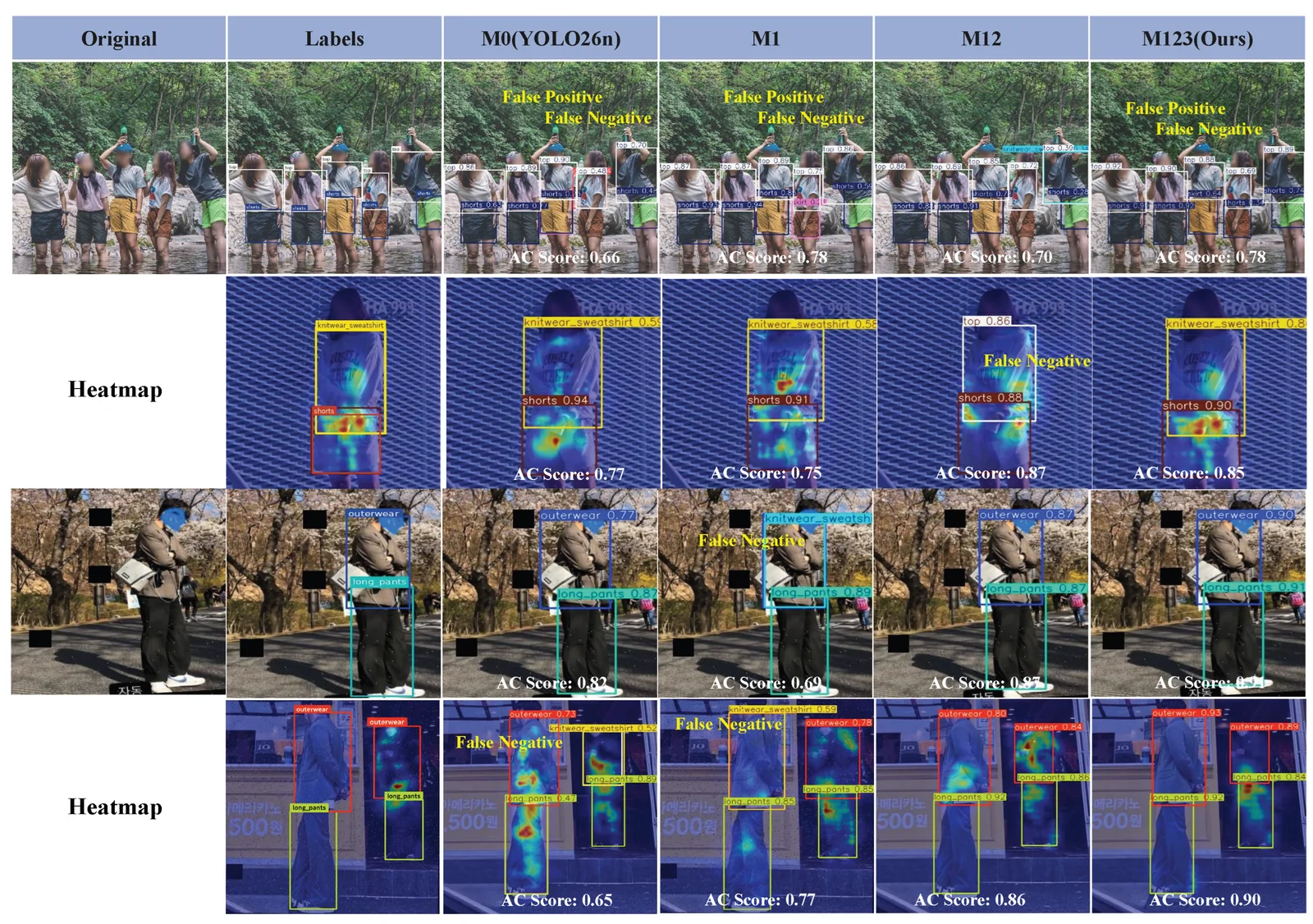
Target-centered qualitative comparison of representative ablation variants. The ground-truth column and four model variants are shown for two examples with explicit garment annotations. AC Score denotes the average confidence score of detected garment instances in each image.

Figure 7 presents two target-centered examples in which the ground-truth garment instances are explicitly shown. Across these examples, the full model recovers annotations that are missed or misclassified by some intermediate variants, qualitatively illustrating the reported ablation trends.

**Figure 8:**
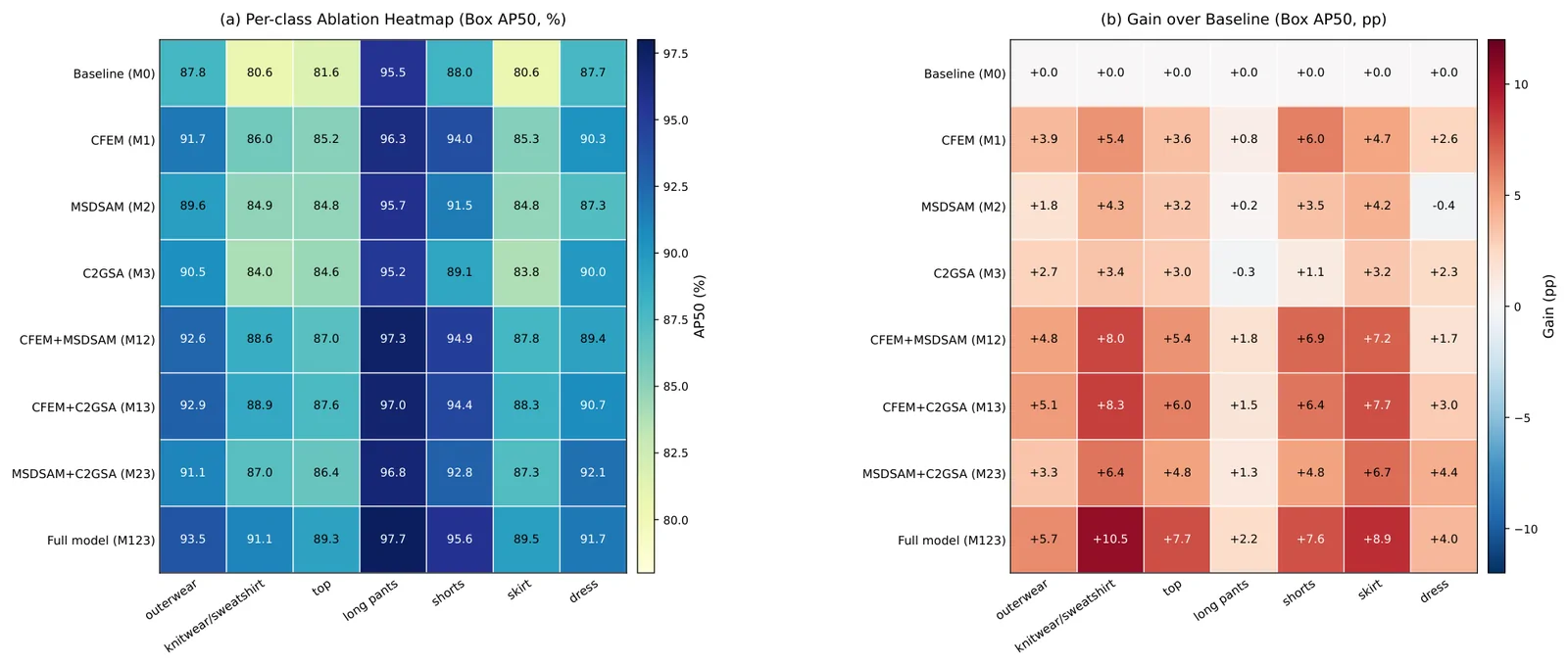
Per-class box AP_50_ ablation results. (**a**) Absolute AP_50_ values for the seven clothing categories. (**b**) Gains relative to the YOLO26n baseline in percentage points.

Figure 8a reports the absolute class-wise AP_50_ values, while Figure 8b isolates the gain over YOLO26n. The largest gains occur for knitwear_sweatshirt, skirt, and top (10.5, 8.9, and 7.7 percentage points), whereas long_pants, which already has the strongest baseline AP_50_, improves by 2.2 points. The two panels therefore show that the aggregate gain is concentrated more strongly in the visually ambiguous categories.

M123 contains 2.39 M parameters and requires 5.4 GFLOPs, representing increases of 0.02 M parameters and 0.2 GFLOPs over the baseline. On the RTX 4090 at 640×640 and batch size 1, throughput is 166 FPS, compared with 175 FPS for the baseline. These values quantify the accuracy–complexity trade-off of the complete configuration.

### 3.2. Cross-Architecture Transferability

To examine whether the proposed modules are specifically dependent on the YOLO26n architecture, C3k2_CFEM, MSDSAM, and C2GSA were further transferred to three representative lightweight YOLO architectures: YOLOv10n, YOLOv12n, and YOLOv13n. These models were selected because they employ different feature representation and aggregation strategies while remaining within the lightweight one-stage detection paradigm.

For each architecture, the proposed modules were mapped to their functionally corresponding stages. C3k2_CFEM was introduced into the main local feature-extraction stages, C2GSA was placed at the high-level semantic representation stage, and MSDSAM was applied before high-resolution prediction to refine multi-scale foreground–background features. The joint integration of all three modules is denoted as “+All”. All variants were trained and evaluated using the same dataset split, input resolution, training settings, and evaluation protocol as the main experiments.

Table 4 evaluates the transferred effects of C3k2_CFEM, MSDSAM, and C2GSA under three backbone and feature-fusion designs. Across YOLOv10n, YOLOv12n, and YOLOv13n, the +All configuration raises mAP_50_ by 3.9, 3.6, and 4.3 percentage points, respectively, while the parameter changes remain within 0.04 M.

### 3.3. Comparison with Representative Object Detectors

The comparison set separates compact general-purpose detectors, which provide the direct accuracy–complexity reference, from broader architectural and clothing-specific references. The general-purpose set includes RT-DETR [38], the open-vocabulary YOLO-World [39], YOLOv6 [40], GhostNet [41], Mamba-YOLO [42], and recent YOLO-family detectors from YOLOv8 to YOLO26 [32,43,44,45,46,47,48,49,50,51,52,53]. Faster R-CNN [54] and the clothing-specific Match R-CNN, Attribute-Mask R-CNN, FashionFormer, and Facere models [3,4,55,56] provide broader architectural and domain-oriented context.

All models use the same dataset split, category taxonomy, 640×640 input resolution, and evaluation metrics. Architecture-specific optimization recipes follow the official or recommended implementations described in Section 2.4. Detection metrics are summarized in Table 5 as means ± standard deviations over five runs.

As shown in Table 5, the proposed model achieves 92.6±0.18% mAP_50_ and 74.7±0.26% mAP_50:95_, exceeding YOLO26n by 6.6 and 5.8 percentage points, respectively. FashionFormer provides the strongest clothing-specific accuracy reference, whereas the lightweight YOLO-family models remain the direct efficiency references. Computational efficiency is examined in Section 3.7.

### 3.4. Class-Wise Detection Performance

To further investigate the category-level behavior of the proposed model, class-wise AP_50_ and AP_50:95_ were compared with those of the original YOLO26n. All results are reported as the means ± standard deviations over repeated runs, as shown in Table 6.

As shown in Table 6, the proposed model improves AP_50_ and AP_50:95_ for all seven categories. The largest gains occur for knitwear_sweatshirt, skirt, and top, with AP_50_/AP_50:95_ increases of 10.5/8.8, 8.9/7.2, and 7.7/6.9 percentage points, respectively.

The dataset contains 7206 long_pants instances and 783 shorts instances. Under the five-run evaluation, shorts reaches 95.6±0.22% AP_50_ and 78.9±0.27% AP_50:95_, compared with 97.7±0.17% and 83.1±0.21% for long_pants. These values document the minority-class result under the imbalanced distribution.

### 3.5. Scale-Conditioned Detection Analysis

To further examine the sensitivity of different detectors to garment scale, an instance-level scale-conditioned evaluation was conducted on the independent test set. For each ground-truth instance, the relative bounding-box area was defined as

(20) $$\rho_{i}=\frac{w_{i}h_{i}}{WH}\times 100\%,$$

where $w_{i}$ and $h_{i}$ denote the width and height of the *i*-th ground-truth bounding box, and *W* and *H* denote the image width and height, respectively.

Scale thresholds were determined from the bounding-box area distribution of the training set. The 25th, 50th, and 75th percentiles were approximately 8.09%, 14.50%, and 25.33%, respectively. For clearer interpretation, these values were rounded to 8%, 15%, and 25% and fixed for all subsequent evaluations. Accordingly, clothing instances were divided into four groups: Small (ρ<8%), Medium (8%≤ρ<15%), Large (15%≤ρ<25%), and Very Large (ρ≥25%).

As shown in Table 7, the four scale groups are relatively balanced in the independent test set, containing 1127, 1154, 999, and 1059 instances, respectively. This distribution provides sufficient samples for evaluating scale-dependent detection behavior while avoiding the instability associated with extremely sparse scale groups.

Predictions were generated only once on the complete test set, and scale-conditioned AP was subsequently computed using a group-specific ignore protocol. For a target scale group *g*, ground-truth instances belonging to *g* were treated as evaluable objects, whereas all ground-truth instances outside *g* were retained in the image but marked as ignored. Predictions were sorted by confidence and matched to ground-truth instances of the same category using the standard one-to-one matching procedure at IoU=0.50, with non-ignored ground truths taking priority over ignored ground truths.

A prediction matched to an in-group ground-truth instance was counted as a true positive. A prediction that could not be matched to an in-group object but matched an out-of-group ground-truth instance was ignored and contributed to neither true positives nor false positives. To determine the treatment of unmatched predictions, the relative area of each predicted bounding box ${\hat{B}}_{k}$ was defined analogously as

(21) $${\hat{\rho }}_{k}=\frac{{\hat{w}}_{k}{\hat{h}}_{k}}{WH}\times 100\%,$$

where ${\hat{w}}_{k}$ and ${\hat{h}}_{k}$ are the width and height of the predicted box. An unmatched prediction was counted as a false positive only when ${\hat{\rho }}_{k}$ fell within the scale interval currently being evaluated; otherwise, it was ignored. Unmatched in-group ground-truth instances were counted as false negatives.

After application of these group-specific ignore masks, the precision–recall curve and AP_50_ were computed using the same class-wise AP implementation as in the overall evaluation, and the resulting AP values were averaged over the seven clothing categories. This protocol prevents objects belonging to other scale groups from being incorrectly penalized while providing an explicit and reproducible assignment rule for unmatched false-positive detections.

Compact general-purpose detectors form the direct conditioned comparison set, while FashionFormer is retained as a clothing-specific accuracy reference.

Here, $\mathrm{Scale}\;\mathrm{Gap}={\mathrm{AP}}_{50}^{\mathrm{Very}\;\mathrm{Large}}-{\mathrm{AP}}_{50}^{\mathrm{Small}}$, where a smaller value indicates lower sensitivity to target-scale variation, and $\mathrm{Small}\;\mathrm{Gain}={\mathrm{AP}}_{50,\mathrm{model}}^{\mathrm{Small}}-{\mathrm{AP}}_{50,\mathrm{YOLO}26n}^{\mathrm{Small}}$.

As shown in Table 8, detection performance generally improves with increasing garment scale, indicating greater difficulty for Small garments with limited spatial detail. The proposed model achieves the highest mAP_50_ in all four scale groups, reaching 89.0%, 92.4%, 94.1%, and 94.9% from Small to Very Large targets, respectively. Its advantage is particularly pronounced for Small garments, where it exceeds YOLO26n by 8.8 percentage points and FashionFormer by 4.1 percentage points.

YOLOv10n, YOLOv12n, and YOLOv13n obtain Small-group mAP_50_ values of 71.8%, 77.9%, and 75.9%, respectively, with Scale Gaps of 13.6, 10.9, and 12.6 points. The proposed model remains the highest-performing method in every scale group.

Moreover, the proposed model exhibits a Scale Gap of 5.9 percentage points, compared with 10.4 points for YOLO26n and 8.7 points for FashionFormer. Under the annotated scale groups and the group-specific evaluation protocol, this result indicates a smaller average performance difference between Small and Very Large instances. Distant or weakly visible garments remain represented among the qualitative failure cases.

This behavior is consistent with the broader small-object detection literature, which attributes scale-dependent degradation to limited visual evidence and increasingly noisy representations [16]. Previous detectors have addressed this problem through feature pyramids or scale-specific receptive fields [17,18]. Recent lightweight YOLO-based studies have further shown that strengthening small-object feature pathways and multi-scale feature fusion can improve detection under limited spatial resolution while maintaining low model complexity [57]. Super-resolution-assisted detectors have also improved small or weak target recognition in orchard, acoustic, and benthic imagery [58,59,60].

### 3.6. Overlap-Conditioned Detection Analysis

In practical clothing images, multiple garment bounding boxes may spatially overlap because of layered dressing, body pose, and crowded appearance. Such configurations increase the ambiguity of adjacent instance boundaries and provide a useful condition for examining detector sensitivity to inter-instance spatial interference. Accordingly, an instance-level overlap-conditioned analysis was conducted on the independent test set.

The available annotations support a geometric bounding-box overlap measure. Visible-region masks or depth-order annotations would be required to quantify the physically occluded fraction of a garment. For two ground-truth garment instances, $B_{i}$ and $B_{j}$, in the same image, their normalized overlap ratio is defined as

(22) $$O_{ij}=\frac{|B_{i}\cap B_{j}|}{\min(|B_{i}|,|B_{j}|)},$$

where $|B_{i}\cap B_{j}|$ denotes the intersection area between the two bounding boxes, and $|B_{i}|$ and $|B_{j}|$ denote their respective areas. Compared with conventional IoU, this formulation is more responsive when a relatively small bounding box is largely contained within a larger neighboring box. The instance-level overlap score is subsequently defined as

(23) $$O_{i}=\max_{j\neq i}O_{ij},$$

with $O_{i}=0$ when no other ground-truth garment instance is present in the same image.

Overlap levels were determined from the training set. The 25th, 50th, and 75th percentiles of $O_{i}$ were approximately 0.0526, 0.1475, and 0.2808, respectively. For clearer interpretation, these values were rounded to 0.05, 0.15, and 0.30 and fixed for all subsequent evaluations. Accordingly, instances were divided into four groups: Low ($O_{i}<0.05$), Mild ($0.05\leq O_{i}<0.15$), Moderate ($0.15\leq O_{i}<0.30$), and Heavy ($O_{i}\geq 0.30$).

As shown in Table 9, the four groups contain 1048, 1160, 1175, and 956 instances in the test set, respectively. The relatively balanced distribution provides sufficient samples for assessing detection behavior under progressively stronger bounding-box overlap.

Predictions were generated once on the original test images. Ground-truth instances were assigned by Equation (23), and unmatched predictions were assigned by the corresponding prediction-side score

(24) $${\hat{O}}_{k\ell }=\frac{|{\hat{B}}_{k}\cap {\hat{B}}_{\ell }|}{\min(|{\hat{B}}_{k}|,|{\hat{B}}_{\ell }|)},\;{\hat{O}}_{k}=\max_{\ell \neq k}{\hat{O}}_{k\ell },$$

where ${\hat{O}}_{k}=0$ when no other candidate prediction is present. The same thresholds of 0.05, 0.15, and 0.30 were applied to ${\hat{O}}_{k}$. The one-to-one matching and group-specific ignore procedures defined in Section 3.5 were retained, with scale intervals replaced by overlap intervals; AP_50_ was computed with the same class-wise implementation and averaged across the seven categories.

The same comparison roles are used in the overlap-conditioned analysis: compact general-purpose detectors provide the direct comparison, and FashionFormer serves as a clothing-specific accuracy reference.

Here, $\mathrm{Overlap}\;\mathrm{Drop}={\mathrm{AP}}_{50}^{\mathrm{Low}}-{\mathrm{AP}}_{50}^{\mathrm{Heavy}}$, where a smaller value indicates lower sensitivity to increasing spatial overlap, and $\mathrm{Heavy}\;\mathrm{Gain}={\mathrm{AP}}_{50,\mathrm{model}}^{\mathrm{Heavy}}-{\mathrm{AP}}_{50,\mathrm{YOLO}26n}^{\mathrm{Heavy}}$.

As shown in Table 10, detection accuracy generally decreases as bounding-box overlap becomes stronger, indicating increased difficulty under stronger inter-instance spatial interference. The proposed model consistently achieves the highest mAP_50_ across all overlap levels, reaching 95.1%, 93.7%, 91.9%, and 88.6% for the Low, Mild, Moderate, and Heavy subsets, respectively.

YOLOv10n, YOLOv12n, and YOLOv13n obtain Heavy-group mAP_50_ values of 70.7%, 76.9%, and 76.2%, with Overlap Drops of 13.5, 11.3, and 11.8 points, respectively. The proposed model remains the highest-performing method in every overlap group.

In the Heavy-overlap subset, the proposed model exceeds YOLO26n by 9.1 percentage points and FashionFormer by 3.6 percentage points. Its Overlap Drop is 6.5 points, compared with 10.8 points for YOLO26n and 8.6 points for FashionFormer. Within this geometric bounding-box protocol, the result indicates a smaller average decline across overlap groups. Severe layered-garment cases remain a documented failure mode in Section 4.2.

Previous studies on crowded object detection have addressed inter-instance interference through repulsion-based localization objectives, occlusion-aware feature extraction, or multi-instance prediction strategies [61,62,63]. Hierarchical locality refinement has similarly been used to recover target evidence under occlusion and inter-instance interference [64]. The smaller Overlap Drop corresponds to a smaller measured decline across the geometric overlap groups. The metric describes bounding-box geometry; physical occlusion requires visible-region or depth-order annotations.

### 3.7. Accuracy–Efficiency Trade-Off

Table 11 reports model complexity and measured inference throughput at 640×640 input resolution and batch size 1. Compact general-purpose detectors provide the direct efficiency reference for the YOLO26n-based model, while two-stage, open-vocabulary, and clothing-specific systems provide broader architectural context. Params, GFLOPs, and FPS characterize model complexity, computational cost, and throughput under the common protocol. The corresponding detection accuracy is listed in Table 5.

Compared with YOLO26n, the proposed model adds 0.02 M parameters (0.8%) and 0.2 GFLOPs (3.8%), while mAP_50_ and mAP_50:95_ increase by 6.6 and 5.8 percentage points. On the RTX 4090 at 640×640 input resolution and batch size 1, throughput decreases from 175 to 166 FPS (5.1%). The measured result is therefore an accuracy-oriented trade-off: higher detection accuracy is accompanied by small increases in model complexity and a moderate throughput reduction.

The clothing-specific models in Table 11 provide domain-oriented accuracy and resource-scale context. Direct efficiency comparison uses the compact general-purpose detectors.

**Figure 9:**
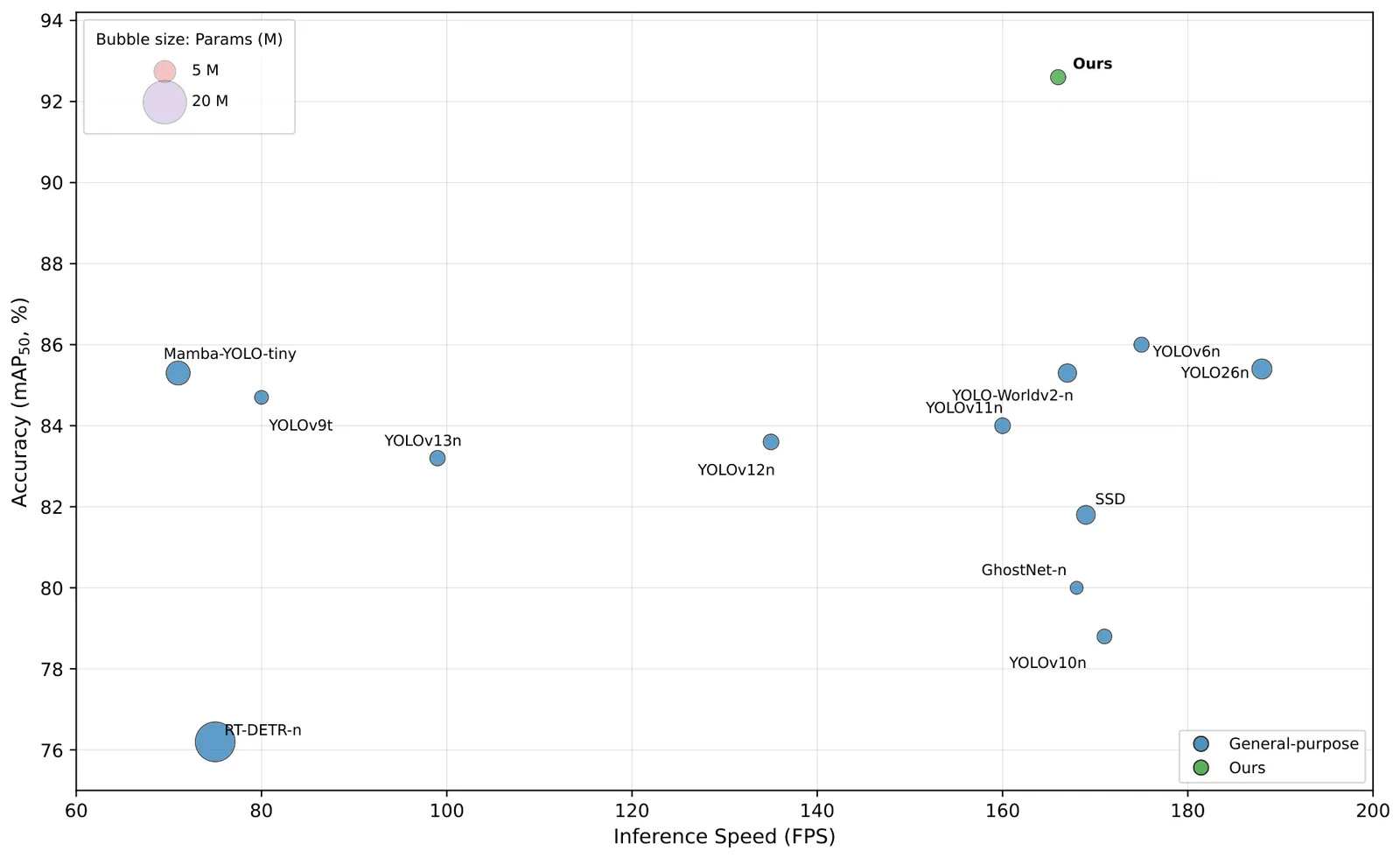
Accuracy–throughput landscape of representative compact general-purpose detectors at 640×640 input resolution and batch size 1. Bubble size represents parameter count.

Figure 9 compares representative compact general-purpose detectors. The proposed model records the highest mAP_50_ among the plotted methods with 2.39 M parameters and 166 FPS. YOLOv10n is smaller and slightly faster at 2.26 M parameters and 171 FPS, while YOLO26n reaches 175 FPS with 2.37 M parameters. Clothing-specific and heavier architectures remain in the tables as domain-oriented accuracy and resource-scale context.

Together with Table 11, the figure shows that the proposed refinement pathway raises mAP_50_ by 6.6 percentage points over YOLO26n with 0.02 M additional parameters, 0.2 additional GFLOPs, and a 5.1% throughput reduction on the RTX 4090.

### 3.8. Cross-Hardware Deployment Analysis

Inference performance was measured on four hardware platforms: an NVIDIA RTX 4090 desktop graphics processing unit (GPU), an RTX 3070 Laptop GPU, an Intel Core Ultra 9 275HX central processing unit (CPU)-only platform, and a Jetson Orin Nano edge device. All models were evaluated on the same test set at 640×640 resolution with batch size 1. The RTX 4090, RTX 3070, and CPU evaluations used the PyTorch implementation, whereas the Jetson evaluation used TensorRT 16-bit floating-point (FP16) inference. FPS is reported as the mean over repeated measurements, and the error bars denote the corresponding standard deviation. Throughput is reported separately for each platform because real-time requirements vary across applications.

**Figure 10:**
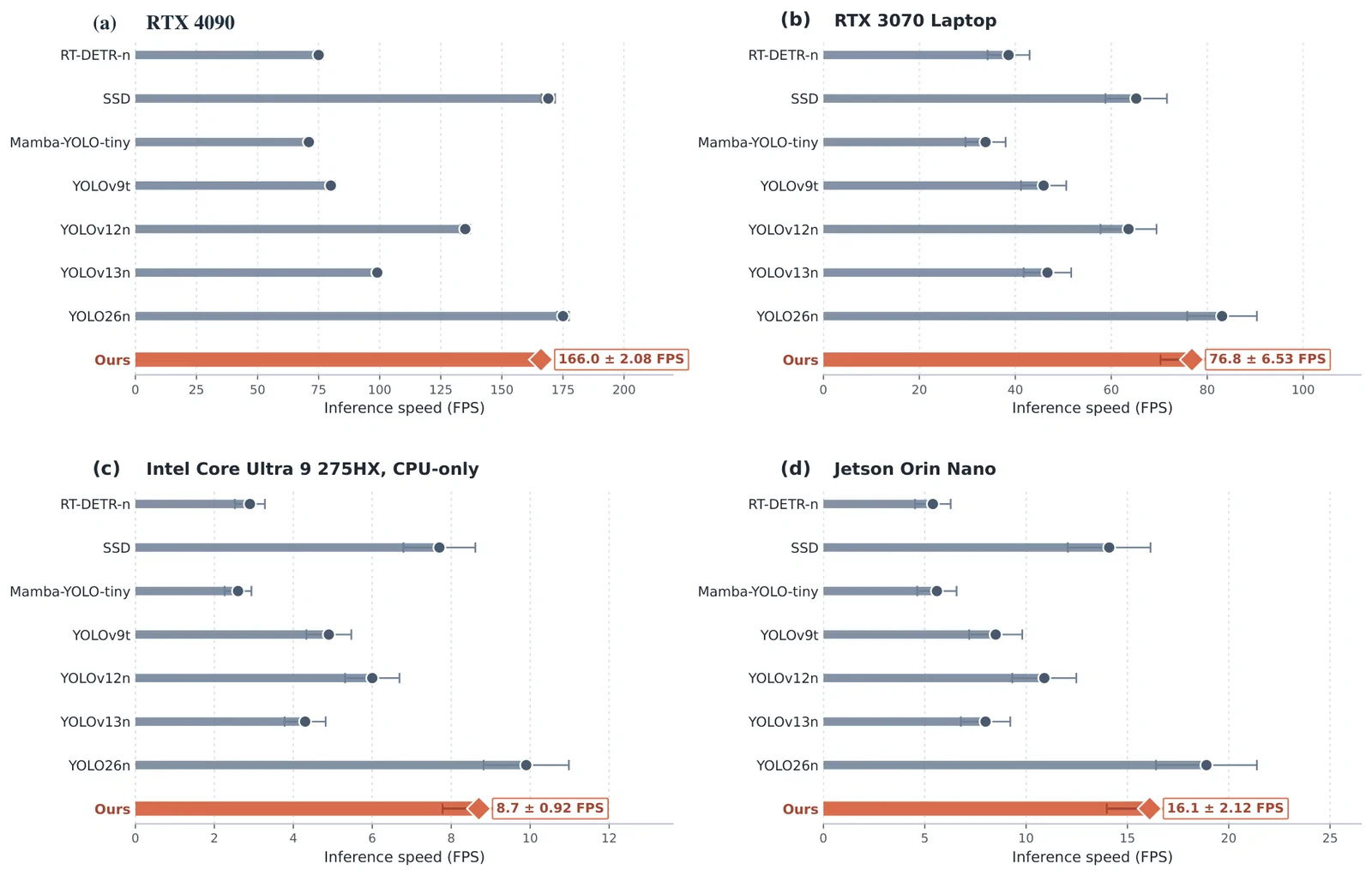
Cross-hardware inference-speed comparison of representative object detectors on four computing platforms: (**a**) RTX 4090, (**b**) RTX 3070 Laptop, (**c**) Intel Core Ultra 9 275HX CPU-only, and (**d**) Jetson Orin Nano. Error bars denote the standard deviation over repeated measurements. Numerical values are displayed only for the proposed model to reduce visual congestion.

Figure 10a–d report 166.0±2.08, 76.8±6.53, 8.7±0.92, and 16.1±2.12 FPS on the RTX 4090, RTX 3070 Laptop GPU, CPU-only platform, and Jetson Orin Nano, respectively. The two GPU platforms provide substantially higher throughput than the CPU and Jetson under the tested settings.

Practical latency also depends on memory access, operator implementation, and platform characteristics, so deployment assessment should jointly consider recognition accuracy, inference throughput, and hardware constraints [65,66,67].

Compared with YOLO26n, the proposed model retains approximately 85–95% of its inference throughput across the four platforms. The relative throughput reduction is larger on the CPU and NVIDIA Jetson Orin Nano (NVIDIA Corporation, Santa Clara, CA, USA) than on the two GPU platforms. These measurements characterize the platform-specific runtime cost of the added refinement operations and motivate hardware-specific optimization for resource-constrained devices.

### 3.9. Robustness Analysis

Common corruptions, including illumination changes, noise, blur, occlusion, and resolution loss, can degrade visual recognition performance [68,69,70,71]. Low-quality video target studies likewise identify camouflage, occlusion, blur, low signal-to-noise ratio, and low resolution as recurring sources of visual difficulty [72]. Nighttime restoration work additionally models haze, illumination, reflectance, and noise jointly [73]. Degradation-aware distillation has recently been used to strengthen object detectors under blur, sensor noise, compression artifacts, and low-resolution capture [74]. Image-restoration-assisted detection provides complementary evidence that recovering degraded visual detail can improve downstream recognition [75]. Physically grounded scattering and illumination adaptation offer another route for handling condition-dependent appearance shifts [76]. Accordingly, five representative perturbations were considered: brightness variation, Gaussian noise, Gaussian blur, random occlusion, and resolution degradation. YOLOv6n, Mamba-YOLO-tiny, YOLO-Worldv2-n, and YOLO26n were selected as comparison models, and mAP_50_ was used as the evaluation metric. The perturbation settings are summarized in Table 12.

For brightness variation, a factor of 1.0 denotes the original illumination condition, while larger and smaller values simulate overexposure and underexposure, respectively. Gaussian noise and Gaussian blur were controlled by the corresponding standard deviation σ. Random occlusion denotes the masked proportion of the target region, whereas resolution degradation was generated by reducing the image resolution according to the specified scale.

**Figure 11:**
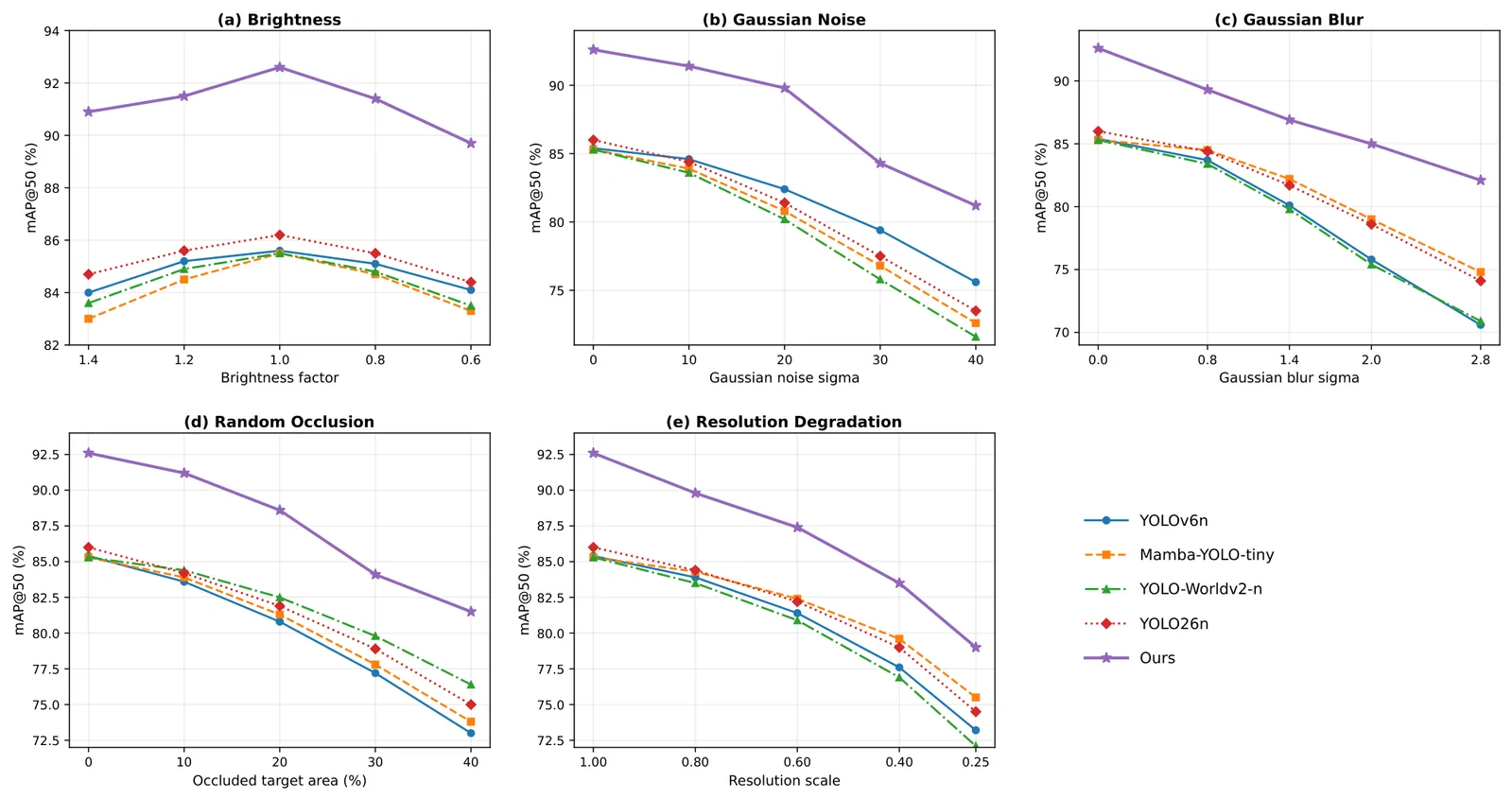
Robustness comparison of different detectors under five perturbation conditions: (**a**) brightness variation, (**b**) Gaussian noise, (**c**) Gaussian blur, (**d**) random occlusion, and (**e**) resolution degradation.

Figure 11a shows that performance peaks near the original illumination condition and decreases under stronger overexposure or underexposure. In Figure 11b, YOLOv6n retains comparatively strong performance as Gaussian noise increases. Figure 11c shows Mamba-YOLO-tiny remaining competitive under Gaussian blur, while Figure 11d shows YOLO-Worldv2-n degrading more slowly under increasing occlusion. In Figure 11e, Mamba-YOLO-tiny remains competitive as resolution decreases. Across the five panels, the proposed model records the highest values at the reported perturbation levels.

Notably, clean-image accuracy and corruption robustness are not necessarily positively correlated across detector architectures [69,77]. The present results similarly reveal perturbation-specific differences among the compared models, while the proposed architecture maintains the strongest aggregate behavior across the tested conditions.

### 3.10. Cross-Source Evaluation

Distribution shifts between training and deployment sources can reduce visual recognition performance [78,79]. A cross-source evaluation was therefore conducted on four publicly available fashion datasets: DeepFashion2 [3], ModaNet [2], Fashionpedia [4], and Exact Street2Shop [80]. These datasets cover commercial platforms, consumer photographs, street-fashion scenes, runway imagery, and uncontrolled real-world environments.

Transferable fusion methods have likewise been used to reduce domain gaps between simulated and real-world collaborative perception settings [81].

External evaluation is performed under two complementary settings. In the zero-shot setting, training and model parameters remain confined to the clothing dataset, followed by direct evaluation on the external datasets. In the retraining setting, each detector is trained on the corresponding external dataset using the same data partition, training budget, and evaluation protocol, while retaining the architecture-specific optimization recipe of its official or recommended implementation. This provides a complementary assessment of target-domain adaptability.

Since the original annotation taxonomies differ among the four datasets, their categories were first harmonized with the seven clothing classes used in this study: outerwear, knitwear_sweatshirt, top, long_pants, shorts, skirt, and dress. Source categories with similar semantic meanings and garment characteristics were merged into the corresponding target class. Only categories that could not be reasonably associated with any of the seven target classes were excluded. This procedure allows all external sources to be evaluated under a consistent clothing taxonomy.

To reduce the influence of dataset scale and ensure a consistent cross-source evaluation budget, 3000 images were selected from each external dataset after category harmonization. The same standardized dataset size was used for all compared detectors. For the retraining setting, each 3000-image subset was further divided into 2100 training, 300 validation, and 600 test images using a fixed 7:1:2 split. Table 13 summarizes the external datasets and the standardized sampling protocol.

The four standardized external datasets therefore provide complementary distribution shifts while maintaining an identical evaluation scale. The following subsections separately report zero-shot transferability and dataset-specific retraining performance.

#### 3.10.1. Zero-Shot Cross-Source Evaluation

To assess strict out-of-domain transferability, all detectors were trained on the clothing dataset and directly evaluated on the four standardized external subsets, each containing 3000 images. Model selection, confidence thresholds, and NMS settings were fixed from the source-domain protocol before external evaluation. The unified seven-class category mapping described above was applied consistently to all models, and mAP_50_ was adopted as the evaluation metric.

As shown in Table 14, the proposed model achieves the highest mAP_50_ on all four external datasets. Its mean mAP_50_ is 73.1%, exceeding YOLO26n and the Best Baseline by 4.5 and 3.5 percentage points, respectively.

#### 3.10.2. Cross-Source Retraining Evaluation

To further evaluate target-domain adaptability, all detectors were retrained separately on the four standardized external datasets after category harmonization. Each dataset contained 3000 images. All compared methods used the same data partition, training budget, and evaluation protocol, while retaining the architecture-specific optimization recipes of their official or recommended implementations. This setting evaluates architecture adaptation with target-domain supervision.

As shown in Table 15, the proposed model obtains the highest mAP_50_ on all four retraining datasets. Its mean mAP_50_ is 86.0%, exceeding YOLO26n and the Best Baseline by 3.6 and 2.1 percentage points, respectively.

## 4. Qualitative Analysis and Discussion

### 4.1. Detection Under Challenging Clothing Scenarios

**Figure 12:**
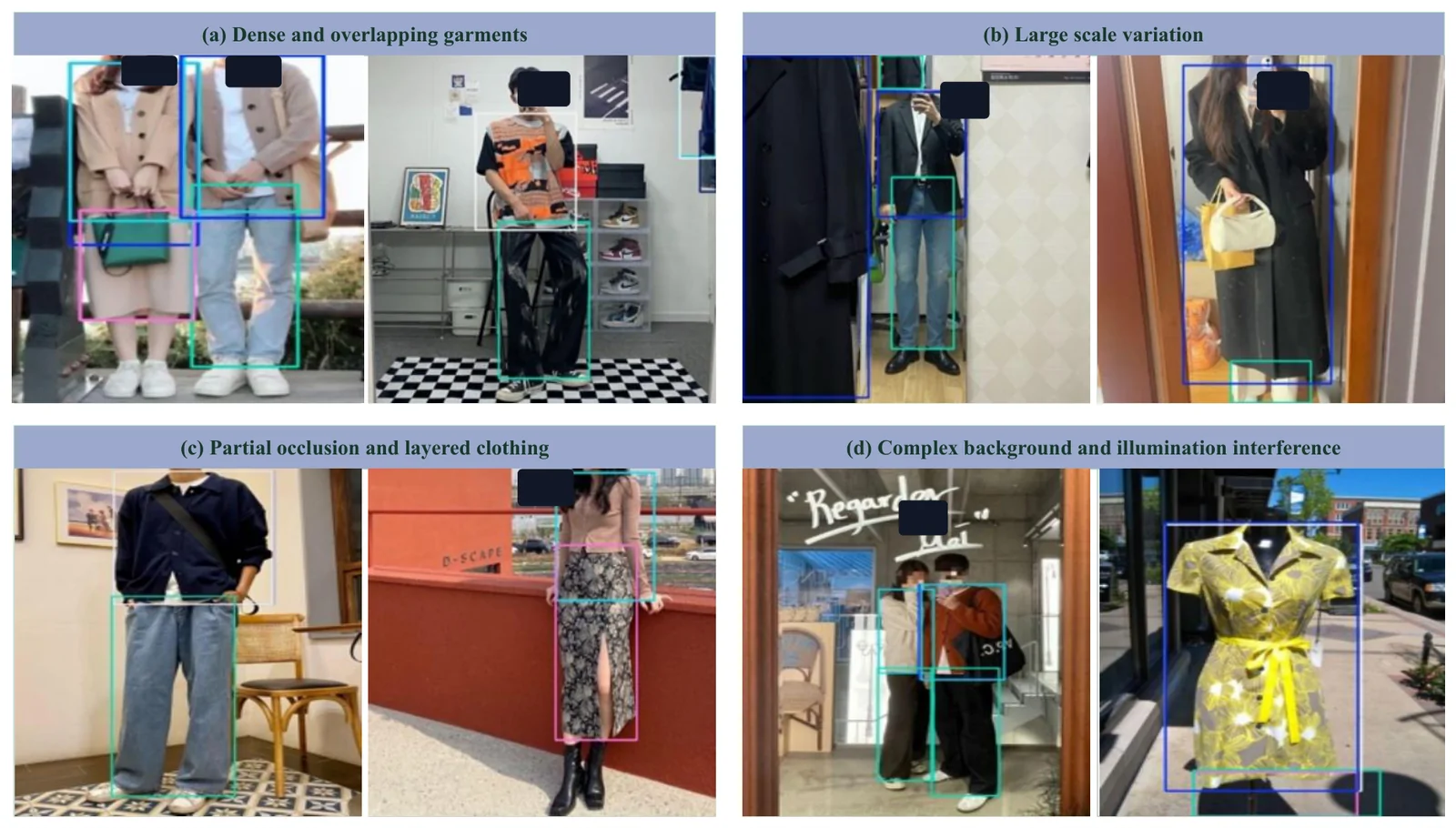
Qualitative detection results under challenging scenarios: (**a**) dense and overlapping garments, (**b**) large scale variation, (**c**) partial occlusion and layered clothing, and (**d**) complex background and illumination interference. Colored bounding boxes indicate detected garments, while black squares mask identifying information. Overlapping boxes reflect the presence of multiple or layered garments.

To assess model behavior under challenging visual conditions, four representative scenarios were selected for qualitative evaluation, including dense and overlapping garments, large scale variation, partial occlusion and layered clothing, and complex background and illumination interference (Figure 12). These examples represent difficult yet successfully detected cases and therefore complement the failure analysis presented later.

Figure 12a shows selected annotated garments that remain separated in a multi-garment scene. Figure 12b contains annotated targets with markedly different spatial extents, and the displayed predictions localize those targets at both scales. Figure 12c illustrates successful detections of partially visible garments using the remaining structural and semantic cues. In Figure 12d, the displayed foreground garments remain localized despite reflections, shadows, vegetation, and cluttered textures.

**Figure 13:**
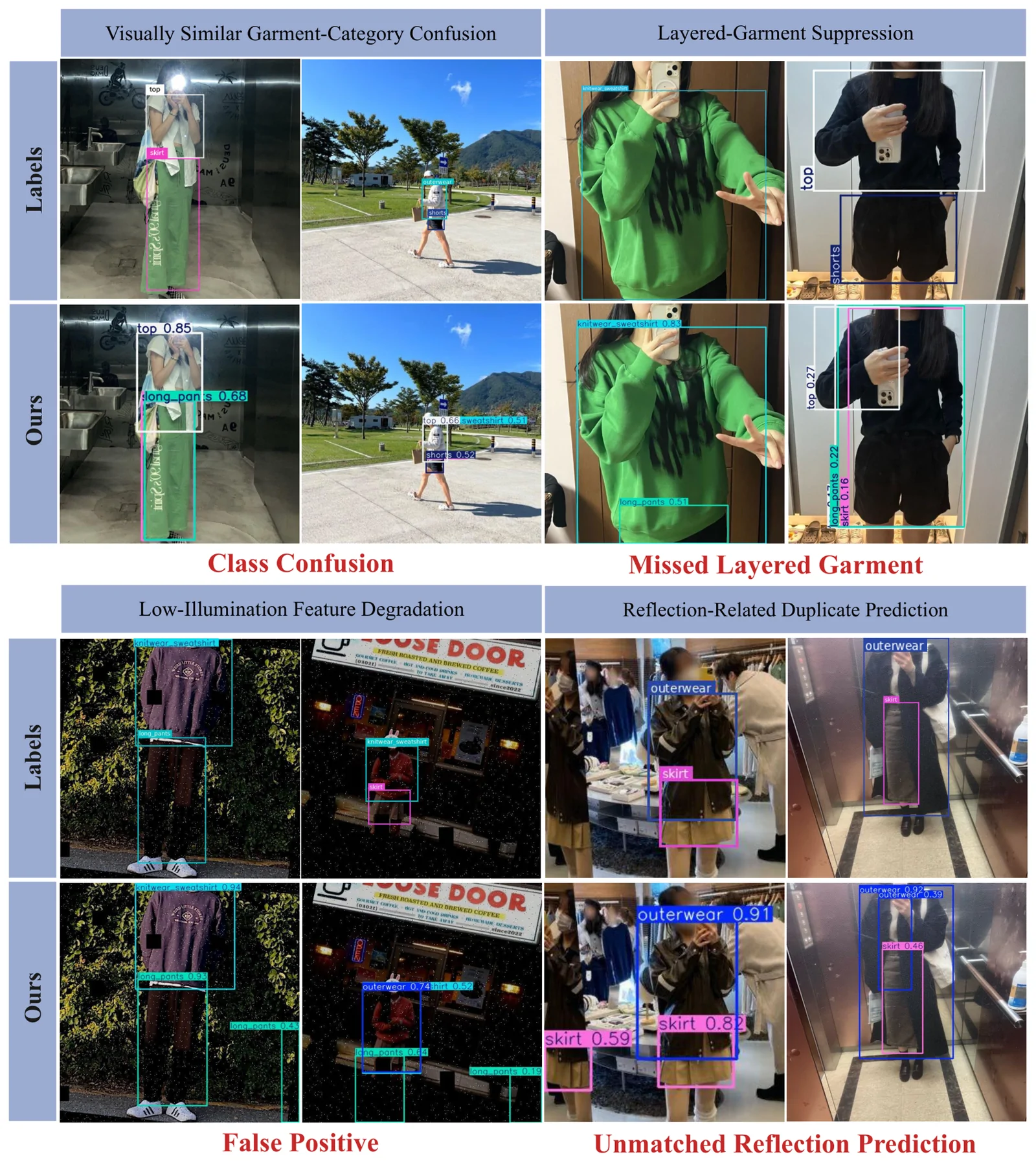
Representative failure cases of the proposed model, including visually similar garment-category confusion (**upper left**), layered-garment suppression (**upper right**), low-illumination feature degradation (**lower left**), and reflection-related duplicate prediction (**lower right**). For each scenario, the upper and lower rows show ground-truth annotations and model predictions, respectively. Colored bounding boxes distinguish garment categories, while black squares mask identifying details. Red text highlights representative detection errors, and predictions on unannotated mirror reflections are identified as unmatched reflection predictions.

These target-centered examples illustrate successful cases under the four displayed conditions and complement the aggregate scale- and overlap-conditioned results and the failure cases in Figure 13. All visible faces are obscured.

### 4.2. Failure Cases and Error Pattern Analysis

Figure 13 shows four recurring failure modes in challenging clothing scenes. These errors involve visually similar category confusion, layered-garment suppression, low-illumination degradation, and reflection-related duplicate prediction.

Clothing categories with similar appearance and structure may lead to incorrect or competing predictions, particularly among visually similar garment types. In layered clothing, severe overlap weakens the features of partially occluded garments, resulting in false positives or missed detections. Under low illumination, reduced contrast and blurred boundaries degrade feature discrimination and increase spurious responses. Reflective surfaces may reproduce garment appearance with sufficient detail to trigger a second prediction. Under the physical-instance annotation protocol, a prediction on the reflected garment remains unmatched. This case captures the ambiguity between high-fidelity reflected evidence and the physical-instance annotation target.

The observed errors are concentrated in cases involving visual category ambiguity and scene-specific interference.

### 4.3. Practical Implications and Limitations

The proposed model provides garment localization and category predictions that can support visual search, automatic garment annotation, intelligent retail, and fashion-oriented image analysis. Cross-hardware evaluation shows lower throughput on the CPU-only and Jetson platforms than on the two desktop/laptop GPU platforms, motivating hardware-specific optimization for resource-constrained deployment.

Recent lightweight systems for greenhouse phenotyping and ecological monitoring illustrate the broader use of compact visual models on field platforms [82,83].

The seven-category taxonomy merges some source-label distinctions, and the class distribution remains imbalanced. Remaining errors concentrate in visually similar, severely truncated, densely interacting, and small-scale garments, while cross-source performance varies with image style, annotation conventions, and category definitions.

## 5. Conclusions

This study proposed a YOLO26n-based clothing detector in which C3k2_CFEM, C2GSA, and MSDSAM form a progressive channel–semantic–spatial refinement pathway. On the reported seven-category dataset, the model achieves 92.6% mAP_50_ and 74.7% mAP_50:95_, improving YOLO26n by 6.6 and 5.8 percentage points with 2.39 M parameters, 5.4 GFLOPs, and 166 FPS on an RTX 4090 at 640×640 input resolution and batch size 1, compared with 175 FPS for YOLO26n.

The scale- and overlap-conditioned results show smaller average degradation than the included comparison methods, while severely layered garments and distant or weakly visible targets remain challenging. Cross-architecture, corruption, cross-hardware, and cross-source evaluations characterize behavior under the tested settings; mean cross-source mAP_50_ is 73.1% in the zero-shot setting and 86.0% after dataset-specific retraining. Overall, the method provides an accuracy-oriented trade-off with 0.02 M additional parameters and 0.2 additional GFLOPs over YOLO26n.

## Figures and Tables

**Table 1:** Experimental environment.

Item	Configuration
Operating system	Ubuntu 22.04
RAM	120 GB
GPU	NVIDIA GeForce RTX 4090
GPU memory	24 GB
CPU	Intel Xeon Platinum 8352V
Deep learning framework	PyTorch 2.1.0
CUDA version	11.8
Python version	3.10.20

**Table 2:** Ablation results of different combinations of the proposed modules. Accuracy metrics are reported as means ± standard deviations over five independent runs using random seeds {0,1,2,3,4}. Params, FLOPs, model size, and FPS are reported as single values.

Model	C3k2_CFEM	MSDSAM	C2GSA	P (%)	R (%)	mAP_50_ (%)	mAP_50:95_ (%)	Params (M)	GFLOPs	Size (MB)	FPS
M0	–	–	–	82.3±0.47	80.1±0.58	86.0±0.34	68.9±0.41	2.37	5.2	5.1	175
M1	✓	–	–	86.5±0.31	83.2±0.44	89.8±0.27	70.9±0.36	2.38	5.3	5.2	169
M2	–	✓	–	84.0±0.52	83.7±0.39	88.4±0.33	70.6±0.48	2.41	5.4	5.2	168
M3	–	–	✓	85.8±0.28	81.8±0.55	88.2±0.29	71.2±0.35	2.34	5.1	5.1	177
M12	✓	✓	–	86.5±0.36	85.8±0.32	91.1±0.21	72.2±0.30	2.42	5.5	5.3	162
M13	✓	–	✓	89.7±0.43	84.1±0.37	91.4±0.24	73.4±0.31	2.35	5.2	5.2	171
M23	–	✓	✓	87.6±0.34	85.0±0.46	90.5±0.30	72.9±0.39	2.38	5.3	5.2	170
M123	✓	✓	✓	91.6±0.29	86.2±0.35	92.6±0.18	74.7±0.26	2.39	5.4	5.3	166

*Note:* Bold values indicate the best detection accuracy for each metric. ✓ and “–” indicate the presence and absence of the corresponding module, respectively.

**Table 3:** $2^{3}$ internal-mechanism factorial ablation. Accuracy metrics are reported as means ± standard deviations over five independent runs.

Variant	ADF	Semantic Gate	Dual Stream	P (%)	R (%)	mAP_50_ (%)	mAP_50:95_ (%)
A0	–	–	–	87.50±0.366	83.70±0.404	89.40±0.290	71.30±0.356
A1	✓	–	–	89.18±0.307	84.17±0.301	90.64±0.389	72.14±0.234
A2	–	✓	–	88.47±0.466	84.61±0.333	89.93±0.358	72.84±0.475
A3	–	–	✓	87.96±0.288	85.14±0.416	90.28±0.298	71.91±0.348
A4	✓	✓	–	90.37±0.428	84.86±0.381	91.28±0.371	73.57±0.270
A5	✓	–	✓	89.76±0.418	85.49±0.391	91.04±0.144	73.11±0.390
A6	–	✓	✓	89.31±0.264	85.91±0.419	91.73±0.307	74.02±0.479
A7	✓	✓	✓	91.60±0.290	86.20±0.350	92.60±0.180	74.70±0.260

*Note:* Bold values indicate the best result for each accuracy metric. ✓ and “–” indicate the presence and absence of the corresponding component, respectively.

**Table 4:** Cross-architecture transferability of C3k2_CFEM, MSDSAM, and C2GSA. “+All” denotes the joint integration of the three proposed modules. All variants use 640×640 inputs.

Architecture	Variant	mAP_50_ (%)	mAP_50:95_ (%)	Params (M)
YOLOv10n	Baseline	78.8	61.3	2.26
	+C3k2_CFEM	80.9	62.5	2.27
	+MSDSAM	80.6	62.9	2.30
	+C2GSA	80.1	63.2	2.24
	+All	82.7	64.6	2.29
YOLOv12n	Baseline	83.6	65.5	2.55
	+C3k2_CFEM	85.0	66.6	2.57
	+MSDSAM	85.9	67.3	2.60
	+C2GSA	85.3	67.0	2.53
	+All	87.2	68.7	2.59
YOLOv13n	Baseline	83.2	64.8	2.46
	+C3k2_CFEM	85.6	66.4	2.47
	+MSDSAM	84.8	66.0	2.52
	+C2GSA	85.0	66.9	2.43
	+All	87.5	68.4	2.50

**Table 5:** Comparison with representative object detectors on the clothing dataset at 640×640 input resolution. Accuracy metrics are reported as means ± standard deviations over five independent runs using random seeds {0,1,2,3,4}.

Model	P (%)	R (%)	mAP_50_ (%)	mAP_50:95_ (%)
RT-DETR-n	77.5±0.53	71.0±0.61	76.2±0.46	58.5±0.57
YOLOv6n	78.3±0.42	78.8±0.36	85.4±0.29	66.9±0.38
GhostNet-n	74.6±0.48	73.7±0.55	80.0±0.37	62.0±0.44
Mamba-YOLO-tiny	79.3±0.35	77.9±0.47	85.3±0.31	67.4±0.40
SSD	80.4±0.44	73.4±0.58	81.8±0.39	60.1±0.52
Faster R-CNN	80.6±0.39	74.3±0.63	83.3±0.34	64.1±0.49
YOLO-Worldv2-n	76.4±0.51	81.5±0.43	85.3±0.28	67.7±0.36
YOLOv9t	80.0±0.37	77.1±0.49	84.7±0.33	68.2±0.42
YOLOv10n	74.3±0.56	70.5±0.62	78.8±0.41	61.3±0.54
YOLOv11n	76.9±0.45	77.7±0.41	84.0±0.30	65.6±0.43
YOLOv12n	79.0±0.32	77.4±0.52	83.6±0.35	65.5±0.39
YOLOv13n	77.9±0.47	75.6±0.38	83.2±0.32	64.8±0.46
YOLO26n	82.3±0.47	80.1±0.58	86.0±0.34	68.9±0.41
Clothing-specific detectors				
Match R-CNN [3]	85.8±0.41	82.6±0.52	87.5±0.33	70.8±0.45
Attribute-Mask R-CNN [4]	87.1±0.36	83.4±0.48	88.4±0.29	71.7±0.39
FashionFormer [55]	89.2±0.31	84.5±0.40	90.1±0.24	73.0±0.32
Facere [56]	87.6±0.37	84.0±0.46	88.8±0.28	72.1±0.37
Ours	91.6±0.29	86.2±0.35	92.6±0.18	74.7±0.26

**Table 6:** Class-wise comparison of AP_50_ and AP_50:95_ between YOLO26n and the proposed model. Results are reported as means ± standard deviations over five independent runs.

Category	YOLO26n AP_50_ (%)	Ours AP_50_ (%)	YOLO26n AP_50:95_ (%)	Ours AP_50:95_ (%)
outerwear	87.8±0.42	93.5±0.27	70.8±0.49	75.8±0.31
knitwear_sweatshirt	80.6±0.56	91.1±0.31	61.9±0.63	70.7±0.38
top	81.6±0.48	89.3±0.24	63.4±0.54	70.3±0.29
long_pants	95.5±0.29	97.7±0.17	80.6±0.33	83.1±0.21
shorts	88.0±0.37	95.6±0.22	72.7±0.46	78.9±0.27
skirt	80.6±0.61	89.5±0.34	62.1±0.67	69.3±0.41
dress	87.7±0.45	91.7±0.28	70.8±0.52	74.8±0.32
Mean	86.0±0.34	92.6±0.18	68.9±0.41	74.7±0.26

**Table 7:** Definition and instance statistics of the scale-conditioned subsets.

Scale Group	Criterion	Train	Val	Test
Small	ρ<8%	3694	522	1127
Medium	8%≤ρ<15%	4021	546	1154
Large	15%≤ρ<25%	3468	534	999
Very Large	ρ≥25%	3849	531	1059
Total	–	15,032	2133	4339

**Table 8:** Instance-level scale-conditioned mAP_50_ (%) comparison of representative clothing detectors. Scale Gap denotes the performance difference between Very Large and Small targets, while Small Gain denotes the improvement over YOLO26n in the Small group. Best and second-best results are highlighted in bold and underlined, respectively. A lower value is preferred for Scale Gap.

Model	Overall	Small	Medium	Large	Very Large	Scale Gap	Small Gain
RT-DETR-n	76.2	69.8	74.9	79.2	81.0	11.2	−10.4
SSD	81.8	75.0	80.3	84.7	87.1	12.1	−5.2
Mamba-YOLO-tiny	85.3	79.9	84.5	87.2	89.4	9.5	−0.3
YOLO-Worldv2-n	85.3	80.7	84.9	87.6	88.2	7.5	+0.5
YOLOv9t	84.7	78.5	83.6	87.1	89.6	11.1	−1.7
YOLOv10n	78.8	71.8	78.6	81.2	85.4	13.6	−8.4
YOLOv12n	83.6	77.9	82.1	87.0	88.8	10.9	−2.3
YOLOv13n	83.2	75.9	82.7	86.8	88.5	12.6	−4.3
YOLO26n	86.0	80.2	85.2	88.1	90.6	10.4	0.0
FashionFormer	90.1	84.9	89.8	92.2	93.6	8.7	+4.7
**Ours**	**92.6**	**89.0**	**92.4**	**94.1**	**94.9**	**5.9**	** +8.8 **

**Table 9:** Definition and instance statistics of the overlap-conditioned subsets.

Overlap Group	Criterion	Train	Val	Test
Low	$O_{i}<0.05$	3663	552	1048
Mild	$0.05\leq O_{i}<0.15$	3927	567	1160
Moderate	$0.15\leq O_{i}<0.30$	4068	594	1175
Heavy	$O_{i}\geq 0.30$	3374	420	956
Total	–	15,032	2133	4339

**Table 10:** Instance-level overlap-conditioned mAP_50_ (%) comparison of representative clothing detectors. Overlap Drop denotes the performance decrease from Low to Heavy overlap, while Heavy Gain denotes the improvement over YOLO26n in the Heavy group. The best and second-best results are highlighted in bold and underlined, respectively. A lower value is preferred for Overlap Drop.

Model	Overall	Low	Mild	Moderate	Heavy	Overlap Drop	Heavy Gain
RT-DETR-n	76.2	80.9	77.8	74.2	69.1	11.8	−10.4
SSD	81.8	86.1	83.4	79.6	74.6	11.5	−4.9
Mamba-YOLO-tiny	85.3	89.1	86.7	83.5	78.8	10.3	−0.7
YOLO-Worldv2-n	85.3	88.2	86.1	83.8	80.1	8.1	+0.6
YOLOv9t	84.7	89.0	85.9	82.6	77.7	11.3	−1.8
YOLOv10n	78.8	84.2	78.9	76.2	70.7	13.5	−8.8
YOLOv12n	83.6	88.2	86.1	81.2	76.9	11.3	−2.6
YOLOv13n	83.2	88.0	84.6	82.4	76.2	11.8	−3.3
YOLO26n	86.0	90.3	87.4	84.2	79.5	10.8	0.0
FashionFormer	90.1	93.6	91.5	89.1	85.0	8.6	+5.5
**Ours**	**92.6**	**95.1**	**93.7**	**91.9**	**88.6**	**6.5**	** +9.1 **

**Table 11:** Model complexity and measured inference throughput on the clothing dataset at 640×640 input resolution and batch size 1. Compact general-purpose detectors provide the direct efficiency reference; other architectures provide broader context.

Model	Params (M)	GFLOPs	FPS
RT-DETR-n	16.75	42.6	75±1.84
YOLOv6n	4.23	11.8	188±3.17
GhostNet-n	1.71	5.0	168±2.46
Mamba-YOLO-tiny	6.01	13.6	71±1.63
SSD	3.68	1.4	169±2.78
Faster R-CNN	28.31	94.8	47±1.21
YOLO-Worldv2-n	3.53	10.1	167±2.14
YOLOv9t	1.97	7.6	80±1.72
YOLOv10n	2.26	6.5	171±2.65
YOLOv11n	2.58	6.3	160±2.31
YOLOv12n	2.55	6.3	135±1.96
YOLOv13n	2.46	6.2	99±1.57
YOLO26n	2.37	5.2	175±2.42
**Ours**	2.39	5.4	166±2.08
Clothing-specific detectors			
Match R-CNN [3]	44.01	112.8	45±1.27
Attribute-Mask R-CNN [4]	46.19	115.9	41±1.32
FashionFormer [55]	43.76	77.3	53±1.43
Facere [56]	45.91	128.1	39±1.14

**Table 12:** Perturbation settings for the robustness experiments.

Perturbation	Level 1	Level 2	Level 3	Level 4	Level 5
Brightness factor	1.4	1.2	1.0	0.8	0.6
Gaussian noise (σ)	0	10	20	30	40
Gaussian blur (σ)	0	0.8	1.4	2.0	2.8
Random occlusion (%)	0	10	20	30	40
Resolution scale	1.0	0.8	0.6	0.4	0.25

**Table 13:** External clothing datasets used for cross-source evaluation.

Dataset	Original Images	Selected Images	Original Classes	Image Domain
I. DeepFashion2 [3]	491,895	3000	13	Commercial and consumer images
II. ModaNet [2]	55,176	3000	13	Street fashion images
III. Fashionpedia [4]	48,825	3000	46	Daily-life, street, runway, and online images
IV. Exact Street2Shop [80]	20,357	3000	11	Real-world street images

**Table 14:** Zero-shot cross-source performance on four external clothing datasets (mAP_50_, %).

Dataset	YOLOv13n	YOLO26n	Best Baseline	Ours	Δ
DeepFashion2	69.4	72.1	73.3	76.2	+2.9
ModaNet	65.9	68.4	69.2	72.4	+3.2
Fashionpedia	61.8	64.9	66.5	70.0	+3.5
Exact Street2Shop	67.2	69.0	69.3	73.6	+4.3
Mean	66.1	68.6	69.6	73.1	+3.5

*Note:* Best Baseline denotes the highest-performing comparison detector on each external dataset, excluding YOLO26n and the proposed model. Δ denotes the improvement of Ours over the Best Baseline.

**Table 15:** Cross-source retraining performance on four external clothing datasets (mAP_50_, %).

Dataset	YOLOv13n	YOLO26n	Best Baseline	Ours	Δ
DeepFashion2	80.8	83.5	84.8	**86.9**	+2.1
ModaNet	81.9	84.7	86.5	**88.2**	+1.7
Fashionpedia	76.5	79.4	81.0	**83.3**	+2.3
Exact Street2Shop	79.0	81.8	83.3	**85.6**	+2.3
**Mean**	79.6	82.4	83.9	**86.0**	**+2.1**

Best Baseline denotes the highest-performing comparison detector on each external dataset, excluding YOLO26n and the proposed model. Δ denotes the improvement of Ours over the Best Baseline.

## Data Availability

The datasets used in this study are available from the cited public sources, and the author-generated annotations are available from the corresponding authors upon reasonable request.
